# The Role of Extracellular Vesicles in the Developing Brain: Current Perspective and Promising Source of Biomarkers and Therapy for Perinatal Brain Injury

**DOI:** 10.3389/fnins.2021.744840

**Published:** 2021-09-24

**Authors:** Teena K. J. B. Gamage, Mhoyra Fraser

**Affiliations:** Department of Physiology, The University of Auckland, Auckland, New Zealand

**Keywords:** extracellular vesicles, exosomes, biomarkers, perinatal brain injury, white matter injury, *in-vivo* animal models, therapeutic strategies

## Abstract

This comprehensive review focuses on our current understanding of the proposed physiological and pathological functions of extracellular vesicles (EVs) in the developing brain. Furthermore, since EVs have attracted great interest as potential novel cell-free therapeutics, we discuss advances in the knowledge of stem cell- and astrocyte-derived EVs in relation to their potential for protection and repair following perinatal brain injury. This review identified 13 peer-reviewed studies evaluating the efficacy of EVs in animal models of perinatal brain injury; 12/13 utilized mesenchymal stem cell-derived EVs (MSC-EVs) and 1/13 utilized astrocyte-derived EVs. Animal model, method of EV isolation and size, route, timing, and dose administered varied between studies. Notwithstanding, EV treatment either improved and/or preserved perinatal brain structures both macroscopically and microscopically. Additionally, EV treatment modulated inflammatory responses and improved brain function. Collectively this suggests EVs can ameliorate, or repair damage associated with perinatal brain injury. These findings warrant further investigation to identify the optimal cell numbers, source, and dosage regimens of EVs, including long-term effects on functional outcomes.

## Perinatal Brain Injury

Preterm infants born <37 weeks gestational age (GA) are at a high risk of brain injury (Penn et al., 2016). Whilst improvements in the survival rates of preterm infants have occurred with advances in obstetric and neonatal care over the past two decades, infants that do survive are likely to experience some degree of long-term neurological impairment. Furthermore, it is the most immature of infants, namely the very preterm infants (28– < 32 weeks GA) and extremely preterm infants (<28 weeks GA) who are at greater risk of lifetime disability and are estimated to account for 5.2% of all preterm births <37 weeks GA (Beck et al., 2010; Blencowe et al., 2012; March of Dimes et al., 2012; Blencowe et al., 2013a).

Cerebral palsy (CP) is the most common adverse neurodevelopmental outcome encountered by this most immature group of preterm infants. CP causes a range of permanent motor disabilities ranging from mild to severe. In the general population, the incidence of CP may be either increasing, static or declining amongst preterm infants (Vincer et al., 2006; Behrman and Butler, 2007; Platt et al., 2007; Robertson et al., 2007; van Haastert et al., 2011; Oskoui et al., 2013). Despite these incongruent trends, subsequent long-term adverse neurodevelopmental outcomes amongst preterm infants who survive constitute a major global health problem and is likely to escalate in the coming years given the increase in maternal age, increased access to assisted reproduction technology, and consequently multiple pregnancies worldwide (Ananth et al., 2005; Yeo et al., 2015; Wang et al., 2017).

Although the pathophysiological mechanisms that trigger injury to the preterm brain are multifactorial and the severity of injury is invariably a consequence of the degree of prematurity, there are several common causes. They include intrauterine infection (i.e., chorioamnionitis), which is recognized as an important factor in the etiology of spontaneous preterm birth and hypoxia-ischemia (HI), including postnatal insults, such as mechanical ventilation-induced lung injury, cerebral blood flow instability, free radical imbalance, sepsis, and necrotising enterocolitis (Stoll et al., 2004; Bassler et al., 2009; Martin et al., 2010; Polglase et al., 2012; Skiöld et al., 2014). Such perinatal insults are intrinsically related to the development of a cascade of central and peripheral inflammatory processes, which play a critical role in the etiology of white matter injury, the most common injury observed in preterm infants. This pattern of brain damage is quite distinctive from that of term infants in that the lesions formed are often confined to the white matter tracts of the periventricular region of the brain, such as the corpus callosum, cingulum bundle, reticular activating system, and superior longitudinal fasciculi (Gopagondanahalli et al., 2016; Murray et al., 2016; van Tilborg et al., 2018). The primary pathological feature of this injury is the formation of focal cystic necrotic lesions and/or diffuse cerebral white matter injury accompanied by astrocytic hypertrophy (gliosis) and microglial activation. This can lead to a loss in white matter volume and impaired myelination, which is evident by magnetic resonance imaging (MRI) (van Tilborg et al., 2018).

While improved neonatal intensive care has resulted in a decline in the incidence of focal cystic necrotic lesions and thus the severest form of injury (Inder et al., 2003a), the incidence of “lesser” neurological morbidity associated with relatively milder forms of white matter injury remains an unresolved clinical issue (Behrman and Butler, 2007; Khwaja and Volpe, 2008; Blencowe et al., 2013b; Back, 2015). Typically, milder white matter injuries have a characteristic pattern featuring noncystic focal or diffuse white matter lesions within the periventricular region and surrounding white matter, which is invariably accompanied by some form of cortical or subcortical gray matter abnormality (Inder et al., 1999; Leviton and Gressens, 2007; Pierson et al., 2007; Keunen et al., 2012; Back and Miller, 2014; Galinsky et al., 2018). Further, converging evidence now suggests that the primary mechanism of myelination failure of the white matter tracts within 24–32 weeks GA, involves loss and subsequent arrested differentiation of oligodendrocyte progenitors, the predominant cell type in human white matter (Volpe et al., 2011; Buser et al., 2012; Back and Miller, 2014; van Tilborg et al., 2016). The more diffuse areas of cerebral white matter injury are characteristically identified as regions where there is a diffuse loss of developing oligodendrocytes and over time, clinically, these lesions are associated with smaller hemispheric size, ventriculomegaly, and impaired gyral development (Rutherford et al., 2010; Skranes et al., 2013; Alexandrou et al., 2014; Engelhardt et al., 2015).

Regardless of whether neurodevelopmental impairments encountered by preterm infants are either severe, moderate, or mild, the impact to the individual is enormous. It is not only a burden to the individual, but also immediate family members, the health care system, and social institutions. The development of effective therapies will enable better outcomes for these infants and thus have significant long-term benefits that could potentially translate to individuals achieving a more productive life including reduced health care costs associated with chronic neurological disability.

Presently, there are limited options for the prevention and/or treatment of perinatal brain injury. Treatment strategies include antenatal administration of magnesium sulfate to women at risk of preterm labor and postnatal administration of erythropoietin to infants born preterm (Leuchter et al., 2014; Juul and Pet, 2015; Crowther et al., 2017). However, these interventions are limited in their effectiveness. In addition, whilst multiple randomized trials suggest that therapeutic hypothermia initiated within the first 6 h of delivery of infants ≥36 weeks GA with suspected moderate or severe hypoxic-ischemic encephalopathy (HIE) for a duration of 72 h, reduces infant morbidity and mortality, it does not completely protect the infant from long-term brain damage (Gluckman et al., 2005; Azzopardi et al., 2009, 2014; Jacobs et al., 2011, 2013; Shankaran et al., 2012; Briatore et al., 2013). Furthermore, preterm infants born <35 weeks GA are not eligible for therapeutic hypothermia despite animal data supporting beneficial effects (Bennet et al., 2007). A pilot study of preterm infants concluded that therapeutic hypothermia was not recommended for preterm infants outside of a clinical research setting given the risk of mortality and side effects (Walsh, 2015). Nevertheless, a randomized controlled trial to assess safety and effectiveness of whole body hypothermia for 72 h in preterm infants 33–35 weeks GA who at <6 h post-delivery exhibit moderate to severe neonatal encephalopathy is currently being conducted (^1^ NCT01793129).

Whilst further randomized clinical trials of hypothermia therapy are warranted amongst preterm infants, there is an ongoing need to develop standalone therapeutic interventions that can be administered to reduce perinatal brain injury or therapeutic interventions that may be administered in conjunction with hypothermia to further reduce infant morbidity and mortality. Indeed, such an approach may be advantageous if the strategy is to target different therapeutic windows and multiple mechanisms of injury. However, hypothermia has the potential to alter the pharmokinetics of adjunct therapies and thus efficacy, which requires careful consideration (van den Broek et al., 2010; de Haan et al., 2012; Lutz et al., 2020).

Stem cells, which are self-renewing and considered the most primordial and least committed cells offer significant promise for treating perinatal brain injury (Kennea and Mehmet, 2004; Santner-Nanan et al., 2005; Vawda et al., 2007; Ikeda, 2008; de Paula et al., 2010; Pimentel-Coelho et al., 2010; Pimentel-Coelho and Mendez-Otero, 2010) given their anti-inflammatory (Li et al., 2005; Murphy et al., 2010; Kim et al., 2012), trophic (van Velthoven et al., 2012; Fu et al., 2017), and regenerative (Ilancheran et al., 2007; van Velthoven et al., 2010; Donega et al., 2014) capabilities. Indeed, neonatal models of hypoxic brain injury using different stem cell types support their therapeutic utility (van Velthoven et al., 2010, 2012; Kim et al., 2012; Donega et al., 2014) and presently there are several adult stroke clinical trials underway (e.g., clinical trials registry numbers: NCT02605707, NCT01151124, NCT04434768, NCT02980354). Despite their clinical potential, very little is known about their risk profile in a clinical neonatal setting. Such considerations are important given their identified potential to elicit unwanted immune responses and tumor formation or emboli (Lappalainen et al., 2008; Fischer et al., 2009; de Almeida et al., 2013).

Substantial evidence suggests that stem cells exert their regenerative effects through the release of biologically active molecules that act in a paracrine manner to promote cell viability and/or proliferation and modulation of immune responses (Baraniak and McDevitt, 2010; Ratajczak et al., 2012). In fact, there has been a growing awareness among scientific and clinical arenas that stem cells are likely to promote neuroprotection and functional recovery via intercellular communication through their release of membrane bound vesicles, referred to as extracellular vesicles (EVs), which have a diverse array of bioactive cargo (Cho et al., 2019b). Therefore, the objective of this unbiased review was to provide an updated evaluation of EVs as therapeutic options in preclinical *in-vivo* animal models of perinatal brain injury.

### EVs

The generic term “EVs” encompasses a heterogeneous range of lipid bilayered particles that are unable to replicate but are secreted by almost every cell of the body (Thery et al., 2018). Historically, EVs have been subdivided into (1) microvesicles (0.1–1 μm in size and often termed ectosomes), which bud from the plasma membrane of cells, (2) nanovesicles (30–150 nm in size and often termed exosomes; in this review they are referred to as “small” EVs), which are generated from late endosomes by inward budding of the limited multivesicular body membrane resulting in the formation of intraluminal vesicles within large multivesicular bodies that are transported to the cell surface and released into the extracellular environment, and (3) apoptotic bodies (1–5 μm in size), which originate from the plasma membranes of apoptotic cells (Pollet et al., 2018). However, there is some overlap in the size of EV subtypes and ambiguity remains between subtypes especially since their underlying intracellular biogenesis pathways cannot always be confirmed (Thery et al., 2018; Mathieu et al., 2019). Consequently, the recently released article “Minimal information for studies of extracellular vesicles 2018 (MISEV2018)” has encouraged researchers to describe EVs based upon (1) physical characteristics (2) biochemical composition, and/or (3) descriptions of conditions or cell of origin to help remove some of the ambiguity between EV subpopulations (Thery et al., 2018). Importantly, EVs carry a range of cargo, including nucleic acids [DNA, RNA, microRNA (miRNA), and non-coding RNAs] (Hunter et al., 2008; Simpson et al., 2008; Kalra et al., 2012; Gezer et al., 2014; Yoon et al., 2014; Abramowicz and Story, 2020; Born et al., 2020), proteins (Simpson et al., 2008; Cai et al., 2013), and lipids (Chen et al., 2019; Nishida-Aoki et al., 2020; Skotland et al., 2020) to local and distal targets that can impact the biology of the target cells by conveying functional properties derived from their cellular source (Cocucci and Meldolesi, 2015; Campanella et al., 2019).

### EV-Mediated Cell Communication and Roles Within the Healthy Brain

All major cell types of the brain, including oligodendrocytes, neurons, microglia, astrocytes, endothelial cells and pericytes secrete EVs (Fevrier et al., 2004; Bakhti et al., 2011; Fitzner et al., 2011; Frühbeis et al., 2013b; Lewis, 2013; Sharma et al., 2013; Prada et al., 2018). A number of stimuli can trigger the release of EVs from neuronal cells. For example, synaptic transmission resulting from calcium influx and glutamatergic synaptic activity elicits secretion of neuron-derived EVs carrying the α-amino-3-hydroxy-5-methyl-4-isoxazolepropionic acid (AMPA) receptor subunit (Budnik et al., 2016). Moreover, glutamate released from neurons triggers EV release from oligodendrocytes through Ca^2+^ entry via oligodendroglial ionotropic glutamate receptors (Frühbeis et al., 2013b).

Although the functional role of the various cell types of EVs within the central nervous system (CNS) is not fully ascertained those identified as being mediated by EV autocrine/paracrine signaling include neural trophic support, synaptic plasticity, regulation of myelination and intercellular communication (Coleman and Hill, 2015; Budnik et al., 2016; Thompson et al., 2016). Neuron-derived EVs have been demonstrated to play a critical role in neurite elongation, a prerequisite step required for the assembly of adult neurons in functional networks (Arantes and Andrews, 2006). Human primary neural cultures treated with human induced pluripotent stem cell-derived neuron EVs (hiPSC-nEVs) results in increased cell proliferation and neuronal cell fate specification and differentiation (Sharma et al., 2019). Additionally, data from preclinical studies, in which hiPSC-nEVs were injected into the lateral ventricle of postnatal day 4 (P4) mice, demonstrate their role in hippocampal neurogenesis (Sharma et al., 2019). Synaptic plasticity is also thought to be mediated by EVs that are known to play roles in synaptic growth (Korkut et al., 2013) and axonal guidance (Gong et al., 2016), as well as synaptic pruning (Bahrini et al., 2015).

Development of the neurocircuitry, is partly mediated through neuronal-EV interactions with glial cells. For example, the uptake of oligodendrocyte derived-EVs by neuronal cells can enhance neuronal cell viability though RAC-alpha serine/threonine-protein kinase (Akt), Extracellular signal-regulated kinases (Erk1/2) and cAMP response element-binding protein (CREB) pathway activation and generate more action potentials suggesting an enhanced potential for neuronal signaling (Fröhlich et al., 2014). Furthermore, the uptake of astrocyte-derived EVs that carry co-chaperone stress-inducible protein 1 (STI1) by neuronal cells can have neurotrophic and neuroprotective roles upon binding to cellular prion protein (PrP^C^) (Hajj et al., 2013). Additionally, dendrite complexity can be enhanced in neurons as a result of the miR-26a-5p cargo of astrocyte-derived EVs (Polanco et al., 2016). The activity of these neuronal networks can also be regulated by microglia-derived EVs that can target neurons where they can act both by stimulating synaptic activity through enhancing sphingolipid metabolism (Emmanouilidou et al., 2010) or inhibiting gamma-aminobutyric acid (GABA)-ergic transmission via N-arachidonoylethanolamine (AEA) signaling (Danzer et al., 2012). Conversely, neuronal-derived EVs can also directly target microglia to stimulate complement C3 activity which aids synaptic pruning (Bahrini et al., 2015).

EVs can also play a significant role in blood-brain barrier (BBB) integrity. Platelet-derived growth factor-BB (PDGF-BB)/PDFG-receptor beta (PDGFR-β) signaling has been implicated in the release of pericyte-derived EVs that carry neuroprotective cargo (Prada et al., 2018). This is particularly relevant in the context of ischemic conditions, since PDGF-BB/PGDFRβ signaling is known to confer protection of the BBB and provide regeneration of infarcted regions by way of enhancing pericyte recruitment (Nakamura et al., 2016). Evidence also suggests that neural progenitor cell-derived EVs enhance post-ischemic BBB integrity by enhancing pericyte recruitment via down-regulation of ATP binding cassette subfamily member 1 (ABCB1) expression and inhibition of the nuclear factor-kappa beta (NF-κβ) pathway and downstream matrix metallopeptidase-9 (MMP-9) activity (Zhang et al., 2021).

Oligodendrocytes, the myelin-forming cells, are well established as having important neuron-protecting roles. Indeed, there is well-founded evidence that oligodendroglial-derived EVs, besides being enriched with myelin proteins [myelin proteolipid protein (PLP), 2′, 3′-cyclic nucleotide 3′-phosphodie- sterase (CNPase), myelin associated glycoprotein, and myelin oligodendroglial glycoprotein (MOG)] are mediators of oxidative stress through transfer of human superoxide dismutase (SOD) and catalase (CAT) (Krämer-Albers et al., 2007). Furthermore, neuronal uptake of oligodendrocyte-derived EVs under *in-vitro* conditions of oxygen and glucose can modulate neuronal firing rate and is associated with enhanced stress resistance and neuronal viability (Frühbeis et al., 2013a; Fröhlich et al., 2014).

The uptake of oligodendrocyte-derived EVs by microglia, the resident macrophage, occurs by way of macropinocytosis, which facilitates the clearance of oligodendrocyte-derived EV cargo and occurs in the absence of microglial activation and without induction of a CD4^+^ T cell response to MOG (Fitzner et al., 2011). However, in pathogenic situations, such as multiple sclerosis, EVs exposed to phagocytic microglia may induce activation of microglia or possibly even evoke signaling to T- and B-cells, which have invaded the CNS via the BBB (Dolcetti et al., 2020). Furthermore, and of particular relevance to the current review, oligodendrocyte-derived EV cargo can act in an autocrine manner to inhibit oligodendrocyte maturation (Bakhti et al., 2011), whereas BBB endothelial cell-derived EVs aid oligodendrocyte precursor survival, motility, and proliferation (Fevrier et al., 2004). Consequently, the clearance of oligodendrocyte-derived EV cargo by microglia in the presence of neuronal cells may allow immature pre-myelinating oligodendrocytes to mature into myelinating oligodendrocytes and thus promote the establishment of myelinated neural circuitry (Bakhti et al., 2011).

**FIGURE 1:**
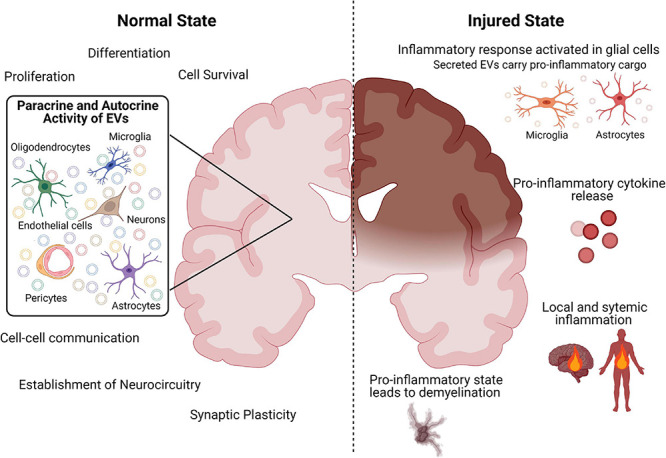
Schematic diagram summarizing the role of EVs in the healthy and injured brain. Created with BioRender.com.

Collectively, the above evidence suggests an intricate, delicate, and highly co-ordinated interplay between EVs and all cell types of the brain that has important implications for the establishment and maintenance of a healthy neural circuitry (Figure 1).

### The Role of EVs in CNS-Immune Communication Following Injury

Over the last two decades, significant emphasis has been placed on understanding the process and timing of injury in the developing brain to improve therapeutic interventions. Seminal studies conducted in animal models have led to the realization that brain injury is an evolving process involving two phases. First there is a latent phase of recovery lasting approximately 6–8 h, followed by a delayed or secondary phase of injury characterized by mitochondrial failure, with seizures and cell swelling that resolves after 72 h (Williams et al., 1991; Fraser et al., 2005, 2007, 2008).

While the etiology of preterm white matter injury is multifactorial, inflammation plays an important role in the pathogenesis of preterm brain injury (Rezaie and Dean, 2002; Deng et al., 2010; Jellema et al., 2013a; Albertsson et al., 2014; Mallard et al., 2014; Dhillon et al., 2015; Hagberg et al., 2015). The ensuing neuroinflammatory cascade following an insult comprises a wide array of both humoral and cellular players. Microglia, the resident macrophage population in the CNS, along with astrocytes, transform into activated cells that migrate or extend their processes, respectively, to sites of injury. Once activated, these cells produce potentially damaging pro-inflammatory mediators, such as pro-inflammatory cytokines [tumor necrosis factor-alpha (TNF-α), interleukin-1β (IL-1β), and interleukin-6 (IL-6)], enzymes and adhesion molecules, including the release of matrix metallopeptidases (MMPs), which lead to the breakdown of the immature BBB (Lau and Yu, 2001; Ranasinghe et al., 2009, 2012; Iadecola and Anrather, 2011; Baburamani et al., 2014). In response to this inflammatory activation, leukocytes migrate through the BBB leaving the brain exposed to systemic inflammatory responses, which further exacerbate injury. Collectively, this results in the progressive destruction of white matter and surrounding CNS tissue.

Modulation of the CNS and peripheral inflammatory response in relation to the timing of the initial insult is most likely key to effective therapeutic targeting. There is a clear recognition from animal models of brain injury that inhibition of EV release from the CNS attenuates systemic responses to CNS inflammation and inhibits BBB leukocyte infiltration, suggesting a damaging role of EVs (Dickens et al., 2017). Indeed, damage-associated molecular pattern (DAMP)-mediated activation with adenosine triphosphate (ATP), results in the release of EVs from microglia (Drago et al., 2017). Specifically, ATP results in modifications of the proteome of EVs derived from microglia responsible for the synthesis of proteins involved in cellular adhesion/extracellular matrix organization, the autophagy-lysosomal pathway and cellular metabolism that can modulate astrocyte activity (Drago et al., 2017). Furthermore, in an IL-1β mouse model of inflammatory brain injury, astrocytic-derived EVs released post-injury are able to induce a systemic inflammatory response in naive animals, in the absence of injury (Figure 1; Dickens et al., 2017).

Microglia-derived EVs appear to play an equally detrimental role in promoting a pro-inflammatory microenvironment response to brain injury (Figure 1). Importantly, recent findings suggest that EVs released from microglia in response to brain injury may represent the major pathway of TNF-α secretion, since EV production is markedly induced by activation of the purinergic receptor P2X7 (P2RX7) by ATP (Bianco et al., 2009). There is also evidence that ATP activation of microglia results in the release of EVs with IL-1β and glyceraldehyde 3-phosphate dehydrogenase (GAPDH) that facilitate the propagation and regulation of the neuroinflammatory response in the brain (Bianco et al., 2005; Takenouchi et al., 2015). In this respect, EVs produced by activated microglia contain high amounts of TNF-α and can induce reactive astrocytic conversion and demyelination (Figure 1; Lombardi et al., 2019).

Clearly redirecting or reprogramming inflammatory glial cells by way of modifying EV signaling toward a beneficial and pro-regenerative function might facilitate repair processes following injury. It would be important also to delineate the origin of EVs in the circulation released in response to injury as they may provide much needed information on possible key facilitators of CNS-peripheral communication and thus identify a specific cellular target for EV therapy.

### Biomarker Potential of CNS-Derived EVs

In addition to the above established and putative roles of EVs within the brain, accumulating evidence suggests that the number and composition of EVs can reflect both healthy and pathological states within the CNS (Fiandaca et al., 2015; Yan et al., 2017; Goetzl et al., 2019; Ohmichi et al., 2019). Although investigations are yet in their infancy, evidence suggests that EVs are found throughout the developing CNS (Morton and Feliciano, 2016) and are likely responsible, at least in part, for normal and pathophysiological brain development in the embryonic and early fetal periods. In support of the former, embryonic cerebrospinal fluid (CSF)-derived small EVs promote neuronal stem cell proliferation possibly through miRNA-mediated regulation of the insulin growth factor pathway (Feliciano et al., 2014; Tietje et al., 2014; Luarte et al., 2016). In addition, small EVs derived from progenitor cells may have protective roles in neuronal development, protecting against such things as hypoxia mediated apoptosis (Deng et al., 2018; Guo et al., 2019; Liu et al., 2019). Furthermore, EVs released by neural stem cells residing in the neonatal subventricular zone (SVZ), lining the lateral ventricles, have the capacity to regulate microglial function as they are enriched with miRNAs (miR-9 and miR-Let7) that regulate microglial morphology (Yao et al., 2014; Morton et al., 2018; Vogel et al., 2018). Indeed, according to novel findings, neonatal SVZ neural stem cell-derived EVs can preferentially target microglia and regulate their morphological and physiological function, suggesting they play important roles within the developing brain (Morton et al., 2018). However, while many of the ascribed EV-mediated effects are foreseen as beneficial they can also be detrimental and under pathological conditions, damaged and distressed cells can release EVs carrying altered protein, lipid, and nucleic acid cargos that may potentially exacerbate the injury (Rajendran et al., 2006; Banigan et al., 2013; Zagrean et al., 2018). Specifically, pathological cells can release EVs that can spread neurological disease and this is most evident in neurodegenerative diseases (Lee and Kim, 2017). For example, cells of Alzheimer’s patients brains are known to release neurotoxic amyloid-β between cells via EVs, which is one mechanism by which disease progression and propagation is thought to occur (Rajendran et al., 2006; Joshi et al., 2014). Similarly, the presynaptic neuronal protein α-synuclein associated with Parkinson’s disease and various other neurodegenerative diseases is transmitted between cells in an EV-mediated manner and drastically impairs neuronal cell viability (Emmanouilidou et al., 2010; Danzer et al., 2012). Moreover, EVs isolated from the CSF of individuals with spinal cord injury and traumatic brain injury express inflammasome proteins, including caspase, apoptosis-associated speck-like protein containing a CARD (ASC), and nod-like receptor protein 1 (NRLP1), suggesting that EVs released in response to CNS damage may be involved in activation of inflammatory signaling processes (de Rivero Vaccari et al., 2016). Consequently, it is equally plausible that EVs released in response to perinatal brain injury may also contribute to the evolution of brain injury pathology over time.

A recently revealed key characteristic of small EVs derived from the fetal CNS is that they are able to cross the fetal BBB and the placental barrier into the maternal circulation making them an attractive source of biomarkers (Sheller-Miller et al., 2016; Goetzl et al., 2019). Adsorptive-mediated transcytosis, translocation and passage from the CSF via arachnoid granulations are all proposed mechanisms of small EV transfer from the CNS into the periphery (Thompson et al., 2016; Matsumoto et al., 2017). However, evidence currently suggests that fetal CNS-derived small EVs do not fuse with the placental membrane in order to cross into the maternal circulation, and further studies are required to clarify the mechanisms of fetal CNS-derived small EV transfer into the maternal circulation (Goetzl et al., 2019). Nevertheless, 20% of total neuronal cell-derived small EVs isolated from first and second trimester maternal plasma samples are comprised of fetal CNS-derived small EVs, which suggests the possibility that their cargo may be possible biomarkers of preterm brain injury (Goetzl et al., 2016).

Investigations have been conducted to assess whether fetal CNS-derived small EVs isolated from maternal plasma during pregnancy can feasibly detect adverse fetal neurodevelopmental outcomes resulting from ethanol exposure as early as the first trimester (Goetzl et al., 2016, 2019). Specifically, the authors of these studies were able to isolate fetal CNS-derived EVs using an antibody raised against Contactin-2/transiently expressed axonal surface glycoprotein-1 (TAG-1), which is expressed transiently on the axonal surface of specific neurons during fetal life (Goetzl et al., 2019). Analysis of fetal CNS-derived EV cargo has revealed that expression of miR-9, a key microRNA involved in neurogenesis (Coolen et al., 2013), and protein levels of synaptic markers neurogranin, synaptotagmin, synatopodin and synaptophysin are all significantly reduced in maternal plasma in association with ethanol exposure (Goetzl et al., 2019). Moreover, levels of neuronal survival proteins [type 1 heat-shock factor (HSF1), B-cell lymphoma extra-large (Bcl-XL) and restriction element-1 silencing transcription factor (REST)] are also significantly lower (Goetzl et al., 2016).

Furthermore, studies conducted to assess whether small EV protein biomarkers are valuable in the diagnosis of brain injury and assessment of the effectiveness of hypothermia have shown that neutral or decreasing fetal neuronal small EV-derived synaptopodin protein levels occurred in maternal plasma of neonates with abnormal neuroimaging scores (Goetzl et al., 2017). Interestingly, evidence from one preterm infant in which CSF-derived small EVs were collected over the course of 4 months following post-hemorrhagic hydrocephalus, there was a reduction in the concentration and proportion of EVs within the 30–100 nm range, but the physiological significance of this is uncertain (Spaull et al., 2019). Together, the studies discussed above are representative of an ever-growing awareness that screening EV profiles of either CSF and plasma of preterm infants or that of their mother’s plasma during pregnancy could be a means to provide early diagnosis of preterm brain injury and ultimately effective implementation of treatment strategies. Furthermore, since EVs can also cross the BBB in the opposite direction (Chen et al., 2016; Mondello et al., 2018; Saint-Pol et al., 2020), their entry into the brain opens up the possibility of harnessing their innate neuroprotective or restorative functions or engineering them for delivery of drugs or biomolecules for the treatment of perinatal brain injury (Cho et al., 2019b).

### EVs as a Therapeutic Agent for Perinatal Brain Injury

*In-vitro* evidence suggests that small EVs have promising neuroprotective and neuroregenerative effects that could be utilized for the treatment of brain injury. Specifically, in a mouse neuroblastoma cell line Neuro-2a model of HI induced by oxygen-glucose deprivation (OGD)/reoxygenation, administration of human umbilical cord Wharton’s jelly MSC-EVs not only resulted in the prevention of HI-induced apoptosis, but also promoted cell survival through EV transfer of miR-let7-5p, a known regulator of caspase-3 (Joerger-Messerli et al., 2018). In addition, researchers recently reported the effect of human Wharton’s jelly MSC-EVs on microglia-mediated neuroinflammation. They observed that MSC-EVs reduced pro-inflammatory cytokine production in response to lipopolysaccharide (LPS) from activated microglia *in-vitro* (Thomi et al., 2019b). Moreover, the treatment of primary rat neurons exposed to thrombin [as a surrogate model of HI with intraventricular hemorrhage (IVH)] with human umbilical cord blood MSC-EVs attenuated cell death (Ahn et al., 2021).

While the majority of *in-vitro* evidence demonstrates EVs from a variety of cell sources have great potential as both neuroprotective and neuroregenerative agents (Doeppner et al., 2015; Jarmalavičiūtė et al., 2015; Bonafede et al., 2016), more information is required from relevant *in-vivo* experimental models of perinatal brain injury before consideration of translation to the clinic. Theoretically, EVs may be biologically safer over cell based therapies as they are potentially less likely to induce an immunogenic response, cause tumor formation, or cause thrombosis in the recipient and in practice easier to collect, store and administer (Adamiak et al., 2018; Ma et al., 2019). However, longitudinal studies are necessary to validate such an assumption. Furthermore, recent data from an *in-vitro* model system demonstrating HI injured neuronal cells potentially can secrete EVs that may contribute to cell death of healthy MSC cells, is of relevance since this could impact on the efficacy of cell based therapies depending on the timing of administration post-injury (Huang et al., 2020).

Current evidence suggests EVs secreted by cells exhibit target selection and that the EV cellular source must be matched to the requirements of the target application (Marcus and Leonard, 2013). In this respect, there is an increasing awareness that generation of neural stem cell-derived EVs would more likely exhibit the highest distribution to the brain and confer neuroprotective benefits. Furthermore, successfully translating interventions from preclinical proof of concept to demonstration of therapeutic value in the clinic requires a route of administration that is minimally invasive, yet effective, such as intranasal delivery, which has a greater likelihood of achieving therapeutic levels. Recent studies of intranasal administration of EVs to neonatal and juvenile rat models of brain injury support the potential clinical relevance of this approach, since EVs targeted a diverse range of brain regions and aggregated to areas of injury (Kodali et al., 2019; Thomi et al., 2019a).

Moving forward, it is apparent the use of well-characterized EVs according to the most recent MISEV guidelines (Thery et al., 2018), produced under specific culture conditions and isolated appropriately to ensure EV integrity, have great promise for treating perinatal brain injury. Consequently, in this unbiased review of the literature the focus was to identify and summarize current evidence of the role of EVs administered to *in-vivo* models of perinatal brain injury as a therapeutic strategy.

### Methods: Search Strategy, Study Selection and Data Extraction

An unbiased search of the literature was conducted using the Preferred Reporting Items for Systematic Reviews and Meta-Analyses (PRISMA) to identify all studies that investigated the potential use of EVs as novel therapeutic agents in animal models of perinatal brain injury (the PRISMA checklist is provided in Supplementary File 1). Eligible studies were identified by searching four databases (PubMed, Embase, Scopus and Web of Science). Keywords included in the search were: perinatal brain injury and extracellular vesicles, perinatal brain injury and exosomes, perinatal hypoxic ischemia and extracellular vesicles, perinatal hypoxic ischemia and exosomes, perinatal encephalopathy and extracellular vesicles, perinatal encephalopathy and exosomes, perinatal brain injury and biomarkers, preterm brain injury and extracellular vesicles, preterm brain injury and exosomes, preterm hypoxic ischemia and extracellular vesicles, preterm hypoxic ischemia and exosomes, preterm encephalopathy and extracellular vesicles, preterm encephalopathy and exosomes, preterm brain injury and biomarkers, fetal brain injury and extracellular vesicles, fetal brain injury and exosomes, fetal hypoxic ischemia and extracellular vesicles, fetal hypoxic ischemia and exosomes, fetal encephalopathy and extracellular vesicles, fetal encephalopathy and exosomes, fetal brain injury and biomarkers, neonatal brain injury and extracellular vesicles, neonatal brain injury and exosomes, neonatal hypoxic ischemia and extracellular vesicles, neonatal hypoxic ischemia and exosomes, neonatal encephalopathy and extracellular vesicles, neonatal encephalopathy and exosomes, and neonatal brain injury and biomarkers. The publication search was conducted on 1 April 2021 and publication dates ranged as far back as 1977. Filters were not applied to any database used in this review of the literature to ensure all relevant papers were captured; instead, articles were manually filtered.

**FIGURE 2:**
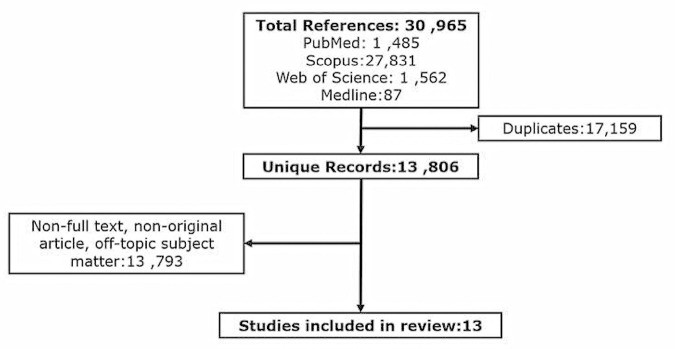
Flow diagram showing the methodical identification and selection of studies investigating the therapeutic potential of EVs to *in-vivo* models of perinatal brain injury.

A total of 30,965 articles were identified and imported into Endnote X7 referencing management software and 17,159 duplicate records removed. The remaining 13,806 articles were analyzed by title and abstract and 13,793 irrelevant articles removed based on non-English, non-full text, non-original article, and off-topic subject matter. Where ambiguity was identified in the title and abstract, the articles were read in full to identify whether the use of EVs as a therapeutic for perinatal brain injury using animal models was investigated. Thirteen articles were identified as relevant for this literature review (Figure 2 and Table 1) based upon this premise.

**TABLE 1:** Investigations of the therapeutic potential of EVs in animal models of perinatal brain injury.

***In-vivo* model**	**EV source**	**EV isolation methods**	**EV Characterization methods**	**Diameter range of isolated EVs**	**Markers investigated in EVs**	**Route, dose, and timing of administration**	**End time points**	**End point measurements**	**Study outcomes**	**References**
P9 or P10 C57BL/6 mouse model of HI treated with either IP or ipsilateral injection of EVs	Human umbilical cord derived MSC conditioned media	Ultracentrifugation	-Nanoparticle tracking analysis -Western blot -TEM	30–100 nm	Membrane markers: CD63+ Endosomal markers: ALIX+ Tsg101+ Cellular makers: GM130-	IP injection 14 h before HI, then immediately before exposure to hypoxia, then immediately after removal of mice from the hypoxic chamber, and finally 3 h post hypoxia Or 2 × 10^5^ cell equivalents of PKH-26 stained EVs transplanted into the ipsilateral hemisphere (1 μL/time) during HI or 6 h post HI	During HI or 6 h post HI	Histology, edema, TTC, behavioral test (neurological severity score), distribution of infrared labeled EVs	1. MSC-EVs reduce edema and cerebral infarction volume associated with HI 2. MSC-EVs can significantly reduce the neurological severity score 3. MSC-EVs help to preserve the structure of neurons at the microscopic level	Han et al., 2020
P9 C57BL/6 mouse model of HI treated with IP injection of EVs	Human bone marrow- derived MSC conditioned media	PEG precipitation followed by ultracentrifugation	-Nanoparticle tracking analysis -Western blot -TEM	108–133 nm	Endosomal markers: Syntenin+ Membrane markers: CD81+, CD9+, CD63+ Cellular markers: calnexin- and prohibitin-	IP injection of 1 × 10^5^ cell equivalents/g bodyweight 1-, 3-, and 5-days post HI	7 days HI	Regional neuropathological scoring, atrophy, histology, RT-PCR	1. MSC-EVs mediate an anti-inflammatory response 2. MSC-EVs stimulate a regenerative response in neurons 3. MSC-EVs stimulate oligodendrocyte maturation	Kaminski et al., 2020
P9 Rice-Vannucci C57/B16 mouse model of HI treated with intranasal administration of EVs	Human bone marrow- derived MSC conditioned media	Ultracentrifugation	-Nanoparticle tracking analysis -Electron microscopy -FACS -Western blot	30–1000 nm	Membrane markers: CD63+ CD81+	Intranasal administration of 6 uL (1.25 × 10^9^ particles/dose) EVs 1 h post HI	Day 2 post EVs treatment	Histology, TUNEL assay, behavioral test (negative geotaxis)	1. MSC-EVs reduced HI induced activation of αMβ2 microglia 2. MSC-EVs reduce HI mediated brain volume loss 3. MSC-EVs decreased cell death and improved behavioral outcomes at day 2 post EV treatment	Sisa et al., 2019
P7 Rice-Vannucci C57/B16 mouse model of HI treated with IC injection of EVs	Mouse bone marrow- derived MSC (either in the presence or absence of miR-21a inhibitor or negative control) conditioned media	Ultracentrifugation followed by size exclusion chromatography (qEV column)	-qNano Gold TRPS -Western blot -TEM	60–160 nm	Membrane markers: CD63+ CD81+ CD9+ Endosomal markers: Tsg101+ Cellular markers: calnexin-	IC injection of 100 ug PKH67-labeled EVs 1 day post HI Or Intracardial IC injection of 100 ug/mL of EVs 1 day post HI	Day 3, 5, 14, 21, 35, 36, 37, 38, 39, 40 post HI	Histology, western blot, brain water content, Nissl, TTC, TUNEL, FISH, TEM, behavioral tests (Morris water maze, Y maze, hindlimb suspension, cliff aversion test)	1. MSC-EVs attenuated acute brain damage and neuroinflammation 2. MSC-EVs stimulated anti-inflammatory populations of microglia and macrophages 3. MSC-EVs reduce neuronal apoptosis 4. MSC-EVs improved injury outcomes in pups 5. MSC-EVs had no effect on long-term memory impairment 6. MSC-EVs miR-21a-5p and TIMP3 cargo are essential for neuroprotective effects	Xin et al., 2020
P7 Rice-Vannucci C57/B16 mouse model of HI treated with IC injection of EVs	Mouse bone marrow- derived MSC (either untreated or pretreated with hydrogen sulfide and in the presence or absence of miR-7b inhibitor or negative controls) conditioned media	Ultracentrifugation followed by size exclusion chromatography (qEV column)	-qNano Gold TRPS -Western blot -TEM	60–380 nm	Membrane markers: CD9+ Endosomal markers: Tsg101+ Cellular markers: calnexin-	IC injection of 100 ug of EVs 1 day post HI	Day 3, 35, 36, 37, 38, 39, and 40, 42 post HI	Western blot, RT-PCR, FACS, TUNEL, Histology TTC, Nissl, edema, FISH, behavioral test (Morris water maze test, novel object recognition test)	1. MSC-EVs (derived from treated or untreated MSC) were present in the ipsilateral hemisphere 2 h post EV treatment and entered both microglia and neurons. 2. H2S-EVs were better at preventing brain tissue loss than untreated EVs 3. H2S-EVs promoted a more anti-inflammatory brain environment 4. H2S preconditioning of EVs was associated with improved long-term cognitive and memory outcomes 5. H2S treatment of MSCs enriches EVs with miR-7b-5p and potentially are responsible for effects observed.	Chu et al., 2020
P7 Sprague Dawley rat model of HI treated with IP injection of EVs	Rat cerebral cortical astrocyte- derived conditioned media	Discontinuous sucrose density gradient combined with ultracentrifugation	-Nanoparticle tracking analysis -Western blot -TEM	110–132 nm	Membrane markers: CD63+ CD81+ Endosomal markers: Tsg101+	IP injection of 2–3 μg of EVs 24 h prior to HI	Day 1, 2, 3, and 7 post HI	Histology, western blot, TTC, TUNEL, ELISA, RT-PCR, oxidative stress, behavioral tests (righting reflex, negative geotaxis reflex, forepaw grip test)	1. Astrocyte-EVs prevent HI brain damage 2. Astrocyte-EVs reduces oxidative stress 3. Astrocyte-EVs carry miR-17-5p cargo that may mediate effects observed.	Du et al., 2021
P4 Sprague-Dawley rat model of IVH treated with ICV injection of EVs	Human umbilical cord blood-derived MSC conditioned media	Ultracentrifugation	-Nanoparticle tracking analysis -Western blot -TEM -SEM	50–100 nm	Membrane markers: CD63+ CD81+ CD9+ Cellular markers: Cytochrome C-, Fibrillarin-, GM130-	ICV injection of 20 ug of EVs two days post IVH	Rat pups monitored daily for the first 7 days post IVH. Subsequent monitoring weekly until P32. MRI at day 7 and day 28 post IVH.	-Histology, MRI, ELISA, behavioral test (negative geotaxis and rotarod test)	1. MSC-EVs performed similarly to MSCs in preventing IVH induced brain injuries, progression of post-hemorrhagic hydrocephalus, and improved behavioral outcomes 2. MSC-EVs mediate neuroprotective activities through BDNF signaling	Ahn et al., 2021
P3 Wistar rat model of LPS/HI treated with IP injection of EVs	Human bone marrow-derived MSC conditioned media	PEG precipitation followed by ultracentrifugation	-Nanoparticle tracking analysis -Western blot	Detail not provided	Endosomal markers: Tsg101+ Membrane markers: CD81+ Microbial: negative for bacteria and viruses	Two repetitive IP injections of EVs (1 × 10^8^ cell equivalents/kg) 3 h prior to and 24 h after IP injection of the vehicle or LPS	Day 2, 8, 27, 87, 122 post EVs treatment	Histology, western blotting, TUNEL assay, cytokine analysis, RT-PCR, diffusion tensor-MRI, behavioral tests (Barnes maze, novel object recognition test, open field test)	1. MSC-EVs reduce inflammation related neuronal degeneration and microgliosis 2. MSC-EVs prevent reactive astrogliosis 3. MSC-EVs prevented myelination defects and white matter damage 4. MSC-EVs treated rats have improved long-term cognitive function compared to untreated controls	Drommelschmidt et al., 2017
P3 Sprague Dawley rat model of white matter damage ***(method unclear)*** treated with ICV injection of EVs	Rat bone marrow- derived MSC conditioned media	Ultracentrifugation	-TEM -Western blot	60–100 nm	Membrane markers: CD63+ Cellular markers: calnexin-	ICV EV injection. Dosage and timing of injection in relation to induction of injury unclear.	Days 1, 3, 5, and 7 post- EVs treatment	Histology, ELISA	1. MSC-EVs enhanced secretion of protective factors 2. MSC-EVs reduced secretion of pro-inflammatory factors 3. MSC-EVs improved prognosis of brain injury	Shu et al., 2018
P3 Wister rat model of LPS/HI treated with intranasal administration of EVs	Human Wharton’s jelly umbilical cord MSC-derived conditioned media	Ultracentrifugation	-TEM -Exo-Check Exosome Antibody array	16–87 nm	Membrane markers: CD63+ CD81+ Flotilin-1+ EpCam+ ICAM+ Endosomal markers: TSG101+ ANXA5+ ALIX+ Cellular makers: GM130-	Intranasal administration of 50 mg/kg of EVs administered at the time of IP LPS injection OR Intranasal administration of 10 mg/kg IRDye^®^ 800CW-labeled EVs administered at the time of IP LPS injection	30 min, 3 h, day 1 and 8, and 4-weeks post LPS/HI	Histology, western blot, RT-PCR, TUNEL assay, distribution of infrared labeled EVs, behavioral tests (Morris water maze)	1. MSC-EVs were detected in frontal region of brain 30 min post intranasal administration 2. MSC-EVs distribute throughout brain at 3 h post-administration 3. Neuron specific cell death is reduced with EV administration prior to ischemia 4. Numbers of oligodendrocytes and neurons were restored in groups treated with MSC-EVs 5. MSC-EV treatment improved learning outcomes	Thomi et al., 2019a
P3 Wister rat model of LPS/HI treated with intranasal administration of EVs	Human Wharton’s jelly umbilical cord MSC-derived conditioned media	Ultracentrifugation	-TEM -Exo-Check Exosome Antibody array	16–87 nm	Membrane markers: CD63+ CD81+ Flotilin-1+ EpCam+ ICAM+ Endosomal markers: TSG101+ ANXA5+ ALIX+ Cellular makers: GM130-	Intranasal administration of 50 mg/kg of EVs administered at time of IP injection of LPS	Day 1 post EVs treatment	Histology, RT-PCR, ELISA	1. MSC-EVs were detected in brain 2. MSC-EVs reduce microglia mediated neuroinflammation	Thomi et al., 2019b
GA d106 fetal sheep model of HI treated with IV injections of EVs	Human bone marrow-derived MSC conditioned media	PEG followed by low-speed centrifugation	-Nanoparticle tracking analysis -Western blot	99–123 nm (ZetaView) and 133–138 nm (NanoSight)	Membrane markers: CD81+ Endosomal markers: TSG101+ Microbial: negative for bacteria, viruses and endotoxins	IV doses of EVs (2.0 × 10^7^ cell equivalents) administered 1 h and 4 days post HI	Day 1 before HI, Day 1, 2, 3, 4, 5, 6, and 7 post HI	Histology, electrophysiological brain parameters	1. MSC-EV treatment reduced number and duration of seizures indicating improved cortical function. 2. MSC-EV treatment preserved baroreceptor reflex indicating improved brain stem function 3. MSC-EV treatment showed a trend only toward protection against hypomyelination (not statistically significant)	Ophelders et al., 2016
GA d106 fetal sheep model of HI treated with IV injections of EVs	Human bone marrow-derived MSC conditioned media	PEG followed by low-speed centrifugation	-Nanoparticle tracking analysis -Western blot -TRPS	Detail not provided	Membrane protein markers: CD81+ Endosomal protein markers: TSG101+	IV doses of EVs (2.0 × 10^7^ cell equivalents) administered 1 h and 4 days post HI	Day 1, 3, and 7 post HI	Histology, western blot	1. MSC-EVs expressing Annexin A1 prevented albumin leakage into the brain indicating maintenance of BBB integrity in injured animals	Gussenhoven et al., 2019

## Results

### Summary of Therapeutic EV Studies in Experimental Animal Models of Perinatal Brain Injury

**FIGURE 3:**
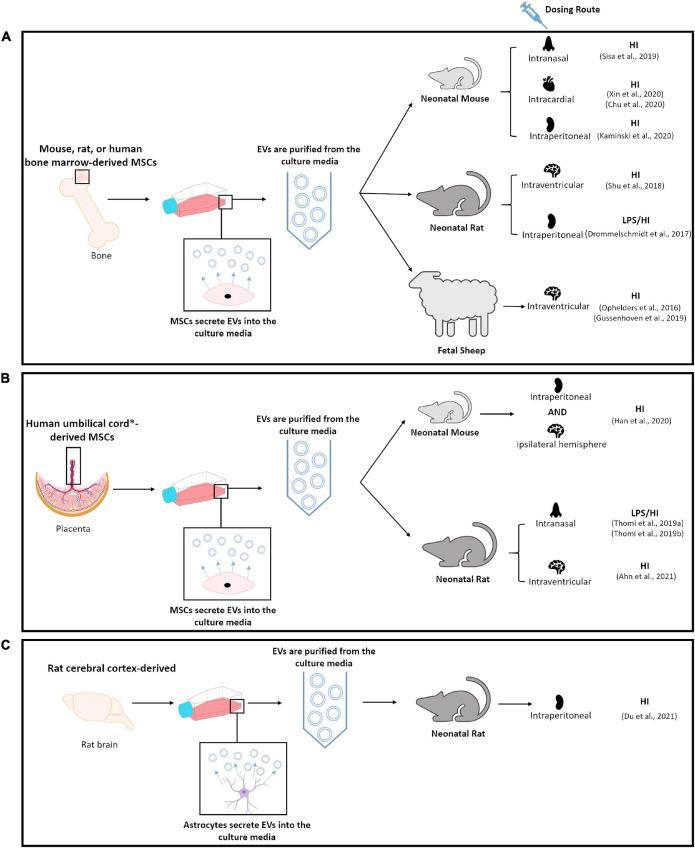
Schematic diagram summarizing methodologies utilized in publications identified by this unbiased review of literature. **(A)** Highlights studies that utilized bone marrow-derived MSCs. **(B)** Highlights studies that utilized umbilical cord^∗^, umbilical cord blood^∗^ or umbilical cord Wharton’s jelly derived MSCs. **(C)** Highlights study which utilized rat cortical-derived astrocytes.

Thirteen studies that utilize EVs as a therapeutic intervention for the treatment of perinatal brain injury using an *in-vivo* model were identified in this unbiased review of the literature (Figure 2 and Table 1; Ophelders et al., 2016; Drommelschmidt et al., 2017; Shu et al., 2018; Gussenhoven et al., 2019; Sisa et al., 2019; Thomi et al., 2019a, b; Chu et al., 2020; Han et al., 2020; Kaminski et al., 2020; Xin et al., 2020; Ahn et al., 2021; Du et al., 2021). Eight of these studies used models of HI alone (Ophelders et al., 2016; Gussenhoven et al., 2019; Sisa et al., 2019; Chu et al., 2020; Han et al., 2020; Kaminski et al., 2020; Xin et al., 2020; Du et al., 2021), three studies used models of simulated infection in the setting of HI (Drommelschmidt et al., 2017; Thomi et al., 2019a, b), one study utilized a model of IVH (Ahn et al., 2021) and one study did not provide sufficient details within the publication to determine the model of brain injury (Shu et al., 2018; Table 1). 12/13 studies utilized MSC-derived EVs. However, 8/12 studies utilized EVs produced by bone marrow-derived MSCs (Ophelders et al., 2016; Drommelschmidt et al., 2017; Shu et al., 2018; Gussenhoven et al., 2019; Sisa et al., 2019; Chu et al., 2020; Kaminski et al., 2020; Xin et al., 2020). Of the eight perinatal brain injury studies which utilized bone marrow-derived MSC-EVs, only one study administered rat MSC-EVs in a rat model (Shu et al., 2018), two studies administered mouse MSC-EVs in a mouse model (Chu et al., 2020; Xin et al., 2020), whereas all other studies utilized human bone marrow-derived MSC-EVs on either a rat (Drommelschmidt et al., 2017), mouse (Sisa et al., 2019; Kaminski et al., 2020) or fetal sheep (Ophelders et al., 2016; Gussenhoven et al., 2019) model (Table 1 and Figure 3). In addition, 2/12 studies utilized human umbilical cord Wharton’s jelly derived MSCs-EVs in a rat model (Thomi et al., 2019a, b), 1/12 studies utilized human umbilical cord blood MSC-EVs in a rat model (Ahn et al., 2021) and 1/12 studies utilized umbilical cord-derived MSC-EVs in a mouse model (Han et al., 2020; Table 1 and Figure 3). Interestingly, one study, which met the eligibility criteria used hydrogen sulfide to pretreat MSCs in order confer a neuroprotective phenotype in MSC-EVs. In this study, hydrogen sulfide treatment enriched the MSC-EVs with miR-7b-5p, a miRNA the authors hypothesized as being responsible for exerting the therapeutic effects of MSC-EVs (Chu et al., 2020). Given the diversity of EV sources utilized in these studies, it warrants mentioning that EVs from different MSC sources may have variable therapeutic potential, since small EVs from human umbilical cord MSCs appear to be most effective in gynecological and perinatal conditions (Cai et al., 2020). Finally, 1/13 studies identified in this literature search utilized rat astrocyte-derived EVs in a neonatal rat model of HI (Table 1 and Figure 3; Du et al., 2021).

In all studies identified, centrifugation (either low speed- or ultra-centrifugation) was employed (either alone or with an additional purification technique) to isolate EVs ranging from 16.34 to 1000 nm (Table 1). Depending on the study, EVs stained positive for a range of membrane protein markers [CD63, CD81, CD9, Flotillin-1, Epithelial cell adhesion molecule (EpCam), and intercellular adhesion molecules (ICAM)] and endosomal markers [Tumor susceptibility gene 101 (TSG101), Annexin A5 (ANXA5), and ALG-2-interacting protein X (ALIX)], and stained negatively for cellular markers (GM130, a cis-Golgi apparatus marker, which monitors cellular contamination of EV isolations, calnexin, an endoplasmic reticulum stress marker, prohibitin and cytochrome C, mitochondrial markers, and fibrillarin, a nucleolar protein) as well as markers of bacteria, viruses and endotoxins (Table 1).

Besides consideration of the optimal source of EVs, the most efficient route of EV administration is also a matter of debate. While intravenous (IV) administration is convenient, it can lead to a high accumulation of EVs in peripheral organs, such as the lung, liver, and spleen (Somiya et al., 2017). However, intranasal administration of therapeutics bypasses peripheral elimination and directly targets the CNS via neural and olfactory pathways innervating the nasal cavity (Merkus and van den Berg, 2007; Hanson and Frey, 2008; Pardridge, 2012). Moreover, researchers consider intranasal administration less invasive and more efficient compared to IV, intracranial, and intra-arterial routes of administration, thus long-term intranasal administration would provide optimal therapeutic application of EVs. However, only 3/13 of the perinatal studies identified in this systemic review have utilized intranasal administration of EVs in their animal models (Figure 3; Sisa et al., 2019; Thomi et al., 2019a, b). Despite the intranasal route offering several advantages, the authors of the other studies identified in this review adopted either intraperitoneal (IP) (Drommelschmidt et al., 2017; Han et al., 2020; Kaminski et al., 2020; Du et al., 2021), IV (Ophelders et al., 2016; Gussenhoven et al., 2019), intracardial (IC) (Chu et al., 2020; Xin et al., 2020), intracerebroventricular (ICV) (Shu et al., 2018; Ahn et al., 2021), or ipsilateral hemisphere (Han et al., 2020) routes of administration of EVs (Figure 3).

Like the mode of delivery, the dosage and duration of delivery also varied between studies. Furthermore, some studies administered EV therapy as a single dose post-injury (Sisa et al., 2019; Thomi et al., 2019b; Chu et al., 2020; Xin et al., 2020; Ahn et al., 2021), whereas others as a repeated dose either prior to and/or following injury (Ophelders et al., 2016; Drommelschmidt et al., 2017; Gussenhoven et al., 2019; Han et al., 2020; Kaminski et al., 2020; Du et al., 2021). There were also differences in the normalization methods of dose; it was either based on a mg/kg body weight (Thomi et al., 2019b), cell equivalents/kg body weight (Ophelders et al., 2016; Drommelschmidt et al., 2017; Gussenhoven et al., 2019; Han et al., 2020; Kaminski et al., 2020), particles/dose (Sisa et al., 2019) or μg of protein (Chu et al., 2020; Xin et al., 2020; Ahn et al., 2021; Du et al., 2021) approach (Table 1). In addition, one study did not provide clear information of the timing and dosage regimen of EVs (Shu et al., 2018).

Clinical presentation of injuries, as well as timing of the initial onset is often unclear and difficult to determine (Ophelders et al., 2020). This is especially apparent in the case of HI injuries, since observed latency for first appearance of seizures can vary from minutes to days following birth (Naeye and Lin, 2001). Despite this limitation, more work is required to determine the most efficacious therapeutic window for initiating EV therapy, since it is more likely that therapeutic interventions delivered, as close to the initial onset of injury, will have a greater chance of enhancing positive long-term neurological outcomes. Equally important, is the need to determine whether time-windows for intervention of EVs can be lengthened.

### EV Therapy Attenuates Microscopic and Macroscopic Damage

Preterm infants, with prior evidence of focal and diffuse white matter lesions, invariably display atypical cortical and subcortical development, involving volumetric reductions in both total cerebral gray and myelinated white matter and larger ventricular volumes either at term or later in childhood (Inder et al., 1999, 2005; Miller et al., 2003). Furthermore, prematurity is frequently associated with a reduction to the size of the corpus callosum, the largest white matter tract within the brain (Schmahmann and Pandya, 2006), which is considered to contribute to later adverse neurodevelopmental outcomes (Caldú et al., 2006). In this context, several preclinical models of perinatal brain injury have shown beneficial effects of EVs in relation to injury-induced deficits in regional volumes and ventricular size of the immature brain.

Based on histological analysis of the Nissl-pattern of staining, intranasal administration of human bone marrow MSC-EVs in a neonatal rat model of HI, significantly reduced mean ipsilateral volume deficits in the pyriform cortex (by 35%), thalamus (by 12.91%), and external capsule (by 10.83%) compared to untreated animals (Sisa et al., 2019). Similarly, neuropathological assessment in cresyl violet stained sections of the neonatal mouse brains collected 7 days after HI and following repeated (1, 3, and 5 days after HI) bolus IP injections of human bone marrow MSC-EVs demonstrated a significant decrease in tissue atrophy within the striatum region compared to both platelet-derived EV (control EVs) and untreated HI controls (Kaminski et al., 2020). Additionally, studies of repeated IP injections of umbilical cord MSC-EVs to neonatal mice both 14 h before HI and immediately before and 3 h following HI, have revealed a significant reduction in edema and cerebral infarction volume compared to untreated HI mice (Han et al., 2020). Further, histological examination of neonatal mice brains 72 h following HI revealed systemic administration of mouse bone marrow MSC-EVs 24 h following HI, attenuated both edema and infarction volume of the ipsilateral hemisphere, as well as cortical tissue loss compared to untreated animals (Xin et al., 2020). However, in some representative coronal sections there was no effect of EV treatment on infarction volume and tissue loss of the hippocampus (Xin et al., 2020). Using the same neonatal mouse model, the authors later tested hydrogen sulfide pretreated MSC-EVs; hydrogen sulfide enriches secreted EVs with miR-7b-5p cargo, an miRNA that has been previously shown to decrease during the first 1-3 days of reperfusion following transient cerebral ischemia in adult rodents (Dharap et al., 2009). Results showed that hydrogen sulfide preconditioning of MSC-EVs, further enhanced their therapeutic ability to reduce cerebral hemispheric infarct volumes and edema (Chu et al., 2020). Moreover, IP administration of rat astrocyte-derived EVs to P7 HI rats similarly reduced infarct volume and also decreased neuronal cell death (TUNEL^+^ cells) compared to untreated HI controls (Du et al., 2021).

Studies conducted by Shu et al. (2018) in which bone marrow MSC-EVs were injected into the lateral ventricle of neonatal rats, hematoxylin and eosin staining showed an absence of cellular edema within areas of the white matter surrounding the lateral ventricle and reduced ventricular enlargement. However, the absence of relevant information as to the nature of the cerebral insult imposed and very limited histopathological assessment performed is a major limitation to this study. Interestingly, Ahn et al. (2021) observed a comparable reduction in ventricular enlargement using serial MRI at P11 and P32 using an IVH rat model treated with human umbilical cord blood-derived MSC-EVs compared to untreated IVH controls. Notably, this effect was not observed in the fibroblast-derived EV controls suggesting this neuroprotective action is specific to MSC-EVs (Ahn et al., 2021).

At the microscopic level, hypomyelination is a central feature of preterm brain injury, which principally affects preoligodendrocytes (or late oligodendroglia progenitors), that predominate in the forebrain at 24–32 weeks gestation (Back et al., 2001; Volpe et al., 2011; Buser et al., 2012; van Tilborg et al., 2016). In addition, such myelination disturbances are considered to arise through depletion of preoligodendrocytes, because of cell death or their subsequent inability to fully differentiate into mature myelinating oligodendrocytes (Back et al., 2001; Volpe et al., 2011; Buser et al., 2012; van Tilborg et al., 2016). Consequently, researchers have sought to investigate the impact that administration of EVs may have in protecting preoligodendrocytes and in turn whether they are able to ameliorate myelination deficits.

Ophelders et al. (2016) investigated the potential of EV therapy to reduce myelination deficits in preterm fetal sheep subjected to umbilical cord occlusion. Fetal systemic administration of human bone marrow MSC-EVs were performed; two boluses of 2.0 × 10^7^ cell equivalents were injected 1 h and 4 days post-umbilical cord occlusion and fetuses killed 7 days thereafter (Ophelders et al., 2016). Treatment improved electrocortical function (namely a decrease in the number and duration of seizures) and a preservation of baroreceptor reflex was associated with partial improvement in myelination as indicated by an increase in the intensity of myelin basic protein (MBP; a marker of mature oligodendrocytes) staining in the subcortical white matter regions of the brain (Ophelders et al., 2016). However, this effect was not paralleled by a reduction in overall cell death, since assessment of apoptotic cell death within subcortical white matter regions revealed no effect of IV EV therapy (Ophelders et al., 2016).

Similarly, in a P2 neonatal rat model of combined inflammation/HI-induced brain injury, intranasal administration of MSC-EVs had no effect on immature and mature oligodendrocyte-specific cell death (TUNEL^+^Olig2^+^ cells) within regions of the corpus callosum following brain injury (Thomi et al., 2019b). However, whilst untreated or vehicle treated neonatal rat models of perinatal brain injury have impaired myelination compared to healthy animals (reduced expression of MBP by 41 and 81% at the gene and protein levels, respectively) (Drommelschmidt et al., 2017; Thomi et al., 2019b), small EV treatment either ameliorated or partially restored myelination (increased expression of MBP by 17 and 38% at the gene and protein levels) following injury (Drommelschmidt et al., 2017; Thomi et al., 2019b). Moreover, long-term follow-up of microstructural white matter changes provided further supportive evidence of the beneficial effect of EVs. Diffusion tensor MRI (DT-MRI) assessment at P125 of P2 rats subjected to LPS/HI-induced brain injury revealed that in EV-treated animals there was increased fractional anisotropy accompanied by reduced radial diffusivity supporting restoration of the microstructure of the corpus callosum (Drommelschmidt et al., 2017). In addition, in a P4 rat model of IVH induced by injection of maternal blood to both lateral ventricles of the brain, ICV administration of MSC-EVs significantly improved myelination 32 days after injury (Ahn et al., 2021). Furthermore, the effect was specific to MSC-EVs since treatment with control fibroblast-EVs was without an effect.

Studies undertaken in the neonatal mouse model of HI also demonstrates that while the number of oligodendrocytes is not impacted by bone marrow-derived MSC-EV treatment, there is enhanced oligodendrocyte maturation and myelination specifically within the striatum and white matter of the external capsule (Kaminski et al., 2020). Of note, treatment with MSC-EVs results in a decrease in the number of the immature O4^+^ oligodendrocytes and an increase in both the number of CC1^+^ differentiated oligodendrocytes and the expression of MBP (Kaminski et al., 2020). Accordingly, this data suggests that MSC-EVs can adversely impact oligodendrocyte differentiation rather than cell numbers.

Like oligodendrocyte precursors, subplate neurons are also selectively vulnerable to degeneration in the preterm brain (Kinney et al., 2012; Back and Miller, 2014; Schneider and Miller, 2019). Intranasal MSC-EV therapy in the P2 neonatal rat model of preterm brain injury, evoked by combined HI/LPS, was recently shown to significantly reduce neuronal cell death (TUNEL^+^NeuN^+^ cells) in the subplate zone of the posterior parietal cortex as well as the CA1 region of the hippocampus (Thomi et al., 2019b). EV therapy also significantly increased both gene and protein expression of microtubule-associated protein 2 (MAP2, a dendritically enriched protein and a marker of synaptic plasticity) in the ipsilateral hemisphere by 19 and 33%, respectively, following injury (Thomi et al., 2019b). Similarly, in the P4 IVH neonatal rat model, TUNEL^+^ cell death was reduced and neurogenesis enhanced upon treatment with MSC-EVs (Ahn et al., 2021). Additionally, therapeutic administration of EVs significantly reduced cell death in the external capsule, cortex and striatum in a mouse model of perinatal bran injury (Sisa et al., 2019; Chu et al., 2020; Kaminski et al., 2020; Xin et al., 2020). Moreover, MSC-EV treatment ameliorated HI-induced ultrastructural characteristics of neuronal cell damage (discontinuous double membrane structures, vacant cytoplasm, swollen mitochondria and reduced nuclear chromatin) (Han et al., 2020; Xin et al., 2020) and improved neural organization (Han et al., 2020). Collectively, the current literature suggests the use of MSC-EVs in preclinical animal models is an attractive alternative to cell-based therapies to restore myelination and neuronal deficits following perinatal brain injury.

### EV Therapy Helps Maintain the Integrity of the BBB

During early human development, the BBB, which protects the brain, was previously viewed as being incomplete. However, evidence has emerged indicating that tight junctional proteins such as claudin-5, occludin, and junctional adhesion molecule (JAM)-1, which are all critical for maintaining the integrity of the BBB are expressed in the germinal matrix, cortex, and white matter of the fetal human brain as early as 16 weeks GA (Ballabh et al., 2005). This suggests that the BBB is functionally developed relatively early in GA, ensuring healthy development of the fetal brain.

Insults to the brain, including HI can lead to a reduction in both the density of blood vessels and a reduction in the functionality of the BBB. For example, in the mouse model of perinatal brain injury there is a significant reduction in vessel density in the striatum compared to sham operated animals (Kaminski et al., 2020). Interestingly this reduction in vessel density is correlated to a reduction in the number of proliferative CD31^+^ endothelial cells (Kaminski et al., 2020). However, treatment with human bone marrow MSC-EVs leads to increased vessel density, particularly within the neurogenic subventricular zone as a result of enhanced proliferation of CD31^+^ endothelial cells (Kaminski et al., 2020).

An additional complication encountered following insults to the brain is the release of free radicals that can cause disruption of the fetal BBB by altering tight junctional molecules (Kumar et al., 2008; Lee et al., 2017). In near-term fetal sheep, free radicals released in response to HI and reperfusion injury can alter expression of tight junctional proteins such as claudin-5, occludin, zonal occludin-1 and zonal occludin-2 (Chen et al., 2012). Furthermore, in preterm fetal sheep, quantification of BBB permeability by assessment of the immunohistochemical distribution of endogenous albumin has revealed that HI-induced leakage of the BBB at day 7 following injury is ameliorated by prior IV administration of two boluses of MSC-EVs (2 × 10^7^ cell equivalents) at 1 h and 4 days following injury (Gussenhoven et al., 2019). Although permeability was not assessed by a fluorescence-based approach, such as IV infusion of FITC-labeled albumin, results suggested that MSC-EV treatment might help to maintain BBB integrity. Additionally, treatment of sham control fetal sheep with MSC-EVs did not incur albumin leakage suggesting that systemic administration of MSC-EVs is a safe therapeutic strategy (Gussenhoven et al., 2019).

### Immunoregulatory and Antioxidant Effects of EV Therapy

A common inflammatory response accompanying the acute degeneration of preoligodendrocytes in non-cystic and diffuse white matter injury is reactive astrocytosis and activated microgliosis (Burda and Sofroniew, 2014). Upon activation, astrocytes and microglia produce a wide array of inflammatory molecules that can have either harmful or beneficial consequences (Hagberg et al., 1996; Kadhim et al., 2001; Baburamani et al., 2014; McNamara and Miron, 2020).

Mesenchymal stem cell-derived EVs are known to have multifaceted immunomodulatory properties and can notably ameliorate inflammatory and apoptotic processes through suppression of pro-inflammatory cytokine production (Ma et al., 2014; Gomzikova et al., 2019), therefore therapeutic administration of MSC-EVs to the injured developing brain may regulate gliosis and other pro-inflammatory mediators. Indeed, in the studies identified by this unbiased review, seven rodent models of perinatal brain injury report a reduction in populations of both astrocytes and microglia in areas of the brain, namely the corpus callosum, cortex, hippocampus and striatum following treatment with MSC-EVs (Drommelschmidt et al., 2017; Sisa et al., 2019; Thomi et al., 2019b; Chu et al., 2020; Kaminski et al., 2020; Xin et al., 2020; Ahn et al., 2021). Of these seven studies, two were conducted in a P3 neonatal rat model of inflammation-induced brain injury reproduced by IP injection of LPS. Combined results from these studies demonstrated that either IP (Drommelschmidt et al., 2017) or intranasal (Thomi et al., 2019b) administration of MSC-EVs attenuated microgliosis (within regions of the corpus callosum, cingulate white matter, and internal capsule) and prevented astrogliosis (within regions of cingulate white matter and internal capsule). Furthermore, attenuation of gliosis was directly associated with a reduction in inflammatory-induced hypomyelination (Drommelschmidt et al., 2017), which is in accordance with evidence of chronic diffuse reactive gliosis within damaged cerebral white matter regions of infants born prematurely (Buser et al., 2012; Back, 2014). Furthermore, findings reported by Drommelschmidt et al. (2017) strengthen the hypothesis that EVs are likely to have no adverse effects, especially since this study underwent a repeated dosing regimen, involving administration of EVs 3 h before and 24 h following LPS injection. These same authors were able to show comparable numbers of astrocytes and microglia within the cortex and white matter between sham-control and sham-control+MSC-EV treated P3 neonatal rats. Similarly, Ahn et al. (2021) demonstrated in a P4 neonatal rat model of IVH, ICV administration of umbilical cord blood-derived MSC-EVs significantly reduced the number of GFAP^+^ astrocytes and ED1-positive microglia, 32 days after injury.

Furthermore, four studies were undertaken in either a P8 (Chu et al., 2020; Xin et al., 2020) or P9 (Sisa et al., 2019; Kaminski et al., 2020) mouse model of term HI, which exhibits predominately gray matter damage (Vannucci and Vannucci, 1997). MSC-EV administration in these studies reduced the numbers of both infiltrating macrophages and brain-resident microglia (Chu et al., 2020; Xin et al., 2020). Specific regional assessment of microglial activation revealed a significant reduction within the cortex, hippocampus and striatum following intranasal, IC, or IP treatment with MSC-EVs (Sisa et al., 2019; Chu et al., 2020; Kaminski et al., 2020; Xin et al., 2020). In addition, MSC-EV administration also appeared to reverse the “ameboid-like” appearance of ionized calcium binding adaptor molecule 1 (Iba1) stained microglia and skewed the ratio of pro-inflammatory M1 macrophages/anti-inflammatory M2 macrophages toward a more anti-inflammatory state compared to untreated HI animals (Xin et al., 2020). It is worth noting that the reduction in microglia within the brains of HI animals was further enhanced through the use of hydrogen sulfide pretreated MSC-EVs with statistically significant differences between untreated MSC-EVs and hydrogen sulfide pretreated MSC-EVs (Chu et al., 2020). IP administration of MSC-EVs appears not to impact the expression of the M1-cell surface marker, CD86, or the M2-cell surface marker, CD206, on microglia post-insult (Kaminski et al., 2020). However, it does reduce the A1-type reactive astrocytic expression of complement C3 protein, which is characteristically upregulated following HI (Hammad et al., 2018; Kaminski et al., 2020) and recognized as having harmful pro-inflammatory and neurotoxic effects (Hammad et al., 2018). Furthermore, it does not impact A2-type reactive astrocytic expression of pentraxin 3 (PTX3), which is developmentally regulated and associated with beneficial anti-inflammatory and pro-regenerative effects (Kaminski et al., 2020).

The above findings are largely in line with those observed by Ophelders et al. (2016) in preterm fetal sheep. They report similar levels of microglial immunoreactivity within subcortical white matter and hippocampal regions between sham-control and sham-control+MSC-EVs fetuses. In the same study, however, MSC-EV treatment failed to mitigate HI-induced microglial activation within the hippocampus. Of concern, MSC-EV treatment exacerbated HI-induced microglial activation within the subcortical white matter, suggesting the neuroprotective mechanism of EV therapy may not involve modulation of inflammatory cells. Interestingly though, splenic weight relative to body weight, which is indicative of activation of a splenic inflammatory response, thus activation of peripheral immune system, was significantly reduced following HI and was restored with MSC-EV administration. However, removal of the spleen also reduces stroke-induced neurodegeneration in an adult rat model, suggesting abatement of the splenic pro-inflammatory response is key to reducing inflammatory-induced cerebral injury (Ajmo et al., 2008; Seifert et al., 2012). Whether the discrepancies observed in modulation of gliosis between these various animal models are a consequence of both the route and timing of EV administration and/or timing of assessment of histopathological outcomes post-insult is unclear.

Aside from the cellular changes observed in response to EV treatment, researchers utilizing rodent models have also examined changes to levels of pro-inflammatory, proapoptotic, and neuroprotective markers during the early stages post-injury. Thomi et al. (2019b) found that at the protein level, MSC-EVs decreased production of pro-inflammatory cytokines TNFα (by 43.6%) and IL-1β (by 42%) in P3 neonatal brain lysates collected 24 h after LPS/HI-induced injury. Presumably, MSC-EVs effects on microgliosis, as noted previously in this study, would have contributed to the down-regulation of pro-inflammatory cytokines. In addition, gene expression profiles of brain lysates revealed MSC-EVs suppressed LPS-induced up-regulation of chemokine (C-X-C motif) ligand-10 (Cxcl10), IL-1β, IL-8 and TNFα transcripts. It is worth mentioning also that in the same study, co-culturing of BV-2 microglial cells with MSC-EVs interfered with TLR4/CD14 signaling pathway, suggesting a mechanism by which EVs mitigate pro-inflammatory production (Thomi et al., 2019b).

In a similar study, Drommelschmidt et al. (2017) observed no effect of MSC-EVs on levels of TNFα and IL-18 mRNA or protein expression in the brain or serum, 48 h after LPS injection. The lack of effect of EVs noted in this study may relate to acute inflammatory responses already being resolved within the 48 h time frame post-LPS injection; however, it could equally reflect other differences such as dosing regimens, routes, and data collection points (Table 1). This is also in contrast to findings reported by Chu et al. (2020) in a mouse model of term HI, where EV therapy attenuated HI-induced mRNA expression of pro-inflammatory cytokines, CD11b, CD32, CD86, cyclooxygenase (COX2), IL-1β, IL-6, inducible nitric oxide synthase (iNOS), and TNFα; an effect which was further enhanced through the use of hydrogen sulfide pretreated MSC-EVs. Similar observations were made by Ahn et al. (2021) in a P4 neonatal rat model of IVH. In their study, both CSF and periventricular brain tissue levels of pro-inflammatory cytokines, IL-1α, IL-1β, IL-6 and TNFα, were significantly reduced following treatment with MSC-EVs compared to both fibroblast-derived EV controls and untreated IVH controls.

Both antenatal and neonatal exposure to endotoxins, such as bacterial LPS, are well documented to trigger innate immunity in the CNS, resulting in microglial activation and oligodendrocyte progenitor and neuronal cell apoptosis in both white and gray matter (Wang et al., 2006). Administration of MSC-EVs significantly reduces apoptosis (caspase-3 protein expression) and cellular degeneration (TUNEL^+^ cells) in both the cortex and white matter in P3 neonatal rats 48 h post LPS injection (24 h post second dose of EVs) (Drommelschmidt et al., 2017). Furthermore, Shu et al. (2018) reported findings to suggest that EVs induce an increase in TGFβ protein expression in association with reduced brain injury. Since anti-apoptotic and anti-excitotoxic effects of TGFβ are well documented, this may represent a mechanism by which EVs reduce the neurodegenerative cascade driven by inflammatory responses. There is conclusive evidence that neuroprotective responses to insults, such as HI, can involve up-regulation of TGFβ, which serves to ameliorate the extent of injury. Notably, however, there are also reports of TGFβ being neurotoxic (Wyss-Coray et al., 1995; Manaenko et al., 2014).

Intuitively, approaches to reduce both oxidative stress and pro-inflammatory damage would be a desirable feature of therapies. IP administration of astrocyte-derived EVs immediately prior to HI, in P7 rats, can effectively reduce brain tissue protein levels of TNF-α and IL-1β and those of oxidative stress factors, SOD, CAT, glutathione peroxidase (GPX) and malondialdehyde (MDA) (Du et al., 2021). Thus, at least, astrocyte-derived EVs appear to have the capacity to promote a neuroprotective environment by way of suppression of oxidative stress- and pro-inflammatory-induced damage.

Taken together, the data reviewed is supportive of the proven anti-inflammatory and anti-apoptotic properties of MSC-EVs (Cho et al., 2019b; Wiklander et al., 2019), and the observed anti-inflammatory and anti-oxidant effects of astrocyte-EVs (Du et al., 2021) are likely to be an underlying principal mechanism that helps to reduce perinatal brain injury.

### EV Therapy Improves Behavioral Functional Outcome

Seven studies have investigated whether EVs repress behavioral impairments associated with perinatal brain injury. Four were conducted in mouse models of term HI (Sisa et al., 2019; Chu et al., 2020; Han et al., 2020; Xin et al., 2020) and four were undertaken in neonatal rat models of preterm HI (Du et al., 2021), LPS/HI (Drommelschmidt et al., 2017; Thomi et al., 2019b) and IVH (Ahn et al., 2021) injury.

Interestingly, in the study where repeated IP injections were conducted of umbilical cord MSC-EVs to neonatal mice 14 h before, immediately before, and 3 h following HI, a significant decline in neurological severity scores for neurological assessment of movement, reflex, sensation, and balance is observed 6 h following HI (Han et al., 2020). Furthermore, in the P9 mouse model of term HI, behavioral negative geotaxis tests conducted at P11, 48 h after HI confirmed that EV treated animals display a significant decrease in the time required to rotate 180° to face uphill after release compared to HI untreated animals (Sisa et al., 2019). Similarly, in the P7 model of term HI, hindlimb suspension tests conducted at P10 and P12 demonstrated that MSC-EV treatment attenuates hindlimb impairment observed in untreated HI animals (Xin et al., 2020). In addition, MSC-EV treatment also reduced the latency to complete the cliff avoidance test suggesting that MSC-EV treatment improves both depth perception and visual impairment post-HI compared to untreated animals (Xin et al., 2020). Moreover, mice treated with MSC-EVs also perform better in the Y-maze test for spatial learning and memory (Xin et al., 2020). Moreover, while untreated HI animals spent less time in the novel arm of the maze compared to sham operated animals, MSC-EV treated HI animals spent more time on the novel arm (Xin et al., 2020). Strikingly, while EV-treated HI animals performed similarly to their untreated counterparts in the novel object recognition test and escape latency tests, HI animals treated with hydrogen sulfide pretreated MSC-EVs had an enhanced performance in both tests (Chu et al., 2020). Finally, MSC-EV treated HI mice also exhibited improved, albeit insignificant, performance during the Morris Water Maze test for long-term memory compared to untreated HI mice (Xin et al., 2020). Their performance was comparable to that of hydrogen sulfide pretreated MSC-EV treated animals (Chu et al., 2020). While the data from the mouse models appears promising, evidence from the four neonatal rat studies is varied.

The study by Drommelschmidt and colleagues was the only study to provide a comprehensive longitudinal follow-up assessment of long-term behavioral outcomes (Drommelschmidt et al., 2017). The authors, however, did not observe any effect of either EV treatment or LPS/HI on learning behavior or motor activity and anxiety-related behavior as assessed by Barnes Water Maze and Open Field tests. Nevertheless, they did suggest that since animals underwent every behavioral test at P30 (adolescent) and P90 (adult), it could have confounded the results (Drommelschmidt et al., 2017). Notably, however, 30 and 90 days after EV administration, they did show an improvement in adaptive memory (Barnes Water Maze test; latency to find the escape hole), and non-spatial and non-aversive memory functions (Novel Object Recognition test; time exploring novel object) compared to animals exposed to LPS/HI alone (Drommelschmidt et al., 2017). Their MRI finding of restoration of white matter microstructure at P125, as previously discussed, agrees well with such improvements in behavioral function following EV treatment.

In later studies, Thomi et al. (2019b) confirmed that in P2 rats, intranasal administration of MSC-EVs following LPS exposure and prior to HI, significantly improved spatial learning performance 4 weeks after brain injury compared to untreated injured animals, as assessed by Morris Water Maze test. Although there was no treatment benefit shown in terms of either short-term or long-term memory performance, their findings of improved myelination together with reduced neuronal cell death in the parietal cortex and hippocampus correlates well with the observed improvements in learning performance (Thomi et al., 2019b).

More recently, Ahn et al. (2021) reported improved behavioral functions following MSC-EV treatment of IVH neonatal rats. They were able to show improvements in balance, coordination, physical condition, and motor-planning using the negative geotaxis (described previously) and the rotarod test, whereby animals were tasked with fall avoidance from a rotating suspended rod. Specifically, they noted the time taken for rats to re-orientate themselves in the negative geotaxis test was shortened when rats were treated with MSC-EVs. These times were comparable to MSC cell treatment of IVH animals, but were notably decreased compared to fibroblast-EV treated animals or untreated IVH controls. The MSC-EV treated animals showed a significant increase (like that of MSC treated animals) in the time spent on the rotating rod compared to fibroblast-EV treated animals or untreated IVH controls.

Finally, neurobehavioral outcomes were also assessed in the neonatal rat model treated IP with rat astrocyte-derived EVs (Du et al., 2021). Assessments were made at 1, 3, and 7 days following HI using the negative geotaxis test (described above), the righting reflex test, where animals are removed from their normal upright position and timed for the reflex which allows them to correctly orientate themselves for brain recovery assessment, and the forepaw grip test, which assesses force and fatigability. Compared to untreated HI control rats, a reduction in both the negative geotaxis response and the righting reflex was observed in rats treated with astrocyte-EVs compared to untreated HI controls. Moreover, forepaw grip was increased in astrocyte-EV treated animals compared to untreated HI controls.

In summary, behavioral studies in rodent models of perinatal brain injury demonstrate that EV treatment not only can improve early neurological deficit scores associated with HI, but also long-term changes in performance of motor coordination (Negative Geotaxis and Rotarod test), spatial learning (Morris Water Maze), adaptive memory (Barnes Water Maze) and non-spatial and non-aversive memory (Novel Object Recognition). Collectively, while this suggests MSC-EVs and astrocyte-EVs have benefits in terms of improving neurobehavioral outcome, more in-depth analyses are needed to resolve contradictory findings that exist between these various studies.

### EV Therapy Improves Electrophysiological Brain Function

Larger animal models, such as the fetal sheep model have the advantage of being able to provide measurements of clinically relevant endpoints for the therapeutic application of EVs against perinatal brain injury that would not otherwise be possible in rodent models. The larger size of fetal sheep facilitates continuous measurement of several physiological variables including electrophysiological brain activity by electroencephalogram (EEG), electrophysiological cardiac function by electrocardiogram (ECG) and hemodynamics by blood pressure and heart rate. In fetal sheep, prolonged umbilical cord occlusion is associated with long-lasting suppression of parasagittal cortical EEG activity, with delayed onset of seizures (Williams et al., 1990; Reddy et al., 1998; Fraser et al., 2005; Lee et al., 2009). In human preterm and term infants, these events predictably correlate with adverse neurological outcomes (Inder et al., 2003b; Murray et al., 2009).

As previously discussed, Ophelders et al. (2016) are presently the only researchers to have conducted a study investigating the therapeutic potential of EVs by assessing electrophysiological brain function in fetal sheep. Although they observed only a modest trend toward protection against HI-induced myelination with IV MSC-EV therapy, which was in contrast to their previous therapeutic study utilizing IV MSC therapy (Jellema et al., 2013b), they showed improved electrocortical function; namely a decrease in the number and duration of seizures. This is consistent with their previous finding demonstrating IV MSC treatment after HI induction reduced seizure activity (Jellema et al., 2013b). An assessment of baroreflex sensitivity (i.e., baroreflex-mediated heart rate) was also undertaken, since dysregulation or impairment of baroreflex function is postulated to augment existing cerebral damage following HI in reperfusion phase (Zwanenburg et al., 2013). IV administration of MSC-EVs to fetuses after HI induction prevented HI-induced impairment of baroreflex reflex sensitivity 3–6 days following HI (Ophelders et al., 2016). However, sham-control MSC-EV treated fetuses displayed impaired baroreflex sensitivity to levels, which was lower than that of fetuses who were subjected to HI alone. This raises real concerns as it may indicate IV administered MSC-EVs may limit the efficacy of the baroreflex-mediated heart rate response to buffer changes in blood pressure. Given the above, further investigations are required to understand these physiological outcomes reported with EV therapy and whether they translate to long-term improvement of cognitive and motor function. Equally important is the need to determine whether differences in dose and timing of administration may alter or further enhance observed beneficial outcomes.

### Candidate Molecular Mediators of EV Therapeutic Activity

While studies identified in this review of the literature suggest MSC-EVs are a useful therapeutic candidate in the treatment of perinatal brain injury, most do not provide a potential mechanism of action through which MSC-EVs exert their effects. There are five exceptions to this, as discussed in detail below.

Two of these papers highlight candidate proteins (Gussenhoven et al., 2019; Ahn et al., 2021). Firstly, the study by Gussenhoven et al. (2019), showing treatment with MSC-EVs helps to maintain BBB integrity in a fetal sheep model of preterm brain injury. They hypothesized that Annexin A1 (an essential endogenous regulator of BBB integrity) expressed on the surface of MSC-EVs interacts with the Annexin A1/Formyl peptide receptor to maintain BBB integrity (Gussenhoven et al., 2019). Moreover, they performed elegant *in-vitro* experiments demonstrating MSC-EVs expressing Annexin A1 or recombinant Annexin A1 improved trans-endothelial electrical resistance (a measure of BBB integrity) of primary fetal rat endothelial cells and that addition of formyl peptide receptor blockers mitigated the effect (Gussenhoven et al., 2019).

Secondly, Ahn et al. (2021) highlight the potential for brain derived neurotrophic factor (BDNF) carried in the cargo of umbilical cord blood-derived MSC-EVs in mediating the neuroprotective effects observed in their P4 neonatal rat model of IVH. Specifically, they demonstrated that the neuroprotective roles of MSC-EVs were attenuated by siRNA transfection of BDNF in MSC-EVs at the macroscopic, microscopic, inflammatory, and behavioral levels. An observation that was not apparent when IVH neonatal rats were treated using the MSC-EVs transfected with random-sequence scrambled siRNA. That BDNF may be a critical neuroprotective mediator is unsurprising given its neuroprotective properties have been identified in a number of neuropathologies including HI (Chen et al., 2013), IVH (Ahn et al., 2017; Ko et al., 2018), meningitis (Bifrare et al., 2005), and traumatic brain injury (Wurzelmann et al., 2017). It is possible that BDNF activation of the tyrosine kinase receptor B (Tkr B), which is highly expressed in the neonatal brain (Webster et al., 2006), may promote neuroprotective and neuroregenerative effects (Chen et al., 2013).

The remaining four highlight candidate miRNAs (Chu et al., 2020; Han et al., 2020; Xin et al., 2020; Du et al., 2021). Firstly, is the study conducted by Xin et al. (2020) in a mouse model of term HI, which showed MSC-EV treatment helped to reduce neuroinflammation, improve neuronal survival and enhance behavioral outcomes. This study used next generation sequencing to profile the miRNA contents of the MSC-EVs used therapeutically (Xin et al., 2020). Using this technique, miR-21a-5p was identified as a potential mediator of the therapeutic effects observed (Xin et al., 2020). Through both *in-vitro* and *in-vivo* assessment it was observed that MSC-EVs enhanced expression of miR-21a-5p, which was significantly reduced in response to HI (Xin et al., 2020). Moreover, the authors proposed that miR-21a-5p may increase both the survival and proliferation of neuronal cells by targeting and reducing the activity of the proapoptotic mediator tissue inhibitor of metalloproteinase 3 (Timp3) (Xin et al., 2020).

Secondly, is the study by Chu et al. (2020), involving pretreatment of MSCs with hydrogen sulfide to generate MSC-EVs enriched with miR-7b-5p. Results suggested miR-7b-5p was responsible for enhancing both the neuroprotective and anti-inflammatory effects of MSC-EVs by binding to the 3′-UTR of the FOS gene. In addition to these candidate molecules, several other miRNAs have been proposed as potential therapeutic mediators and it is possible that they are delivered via EVs and could be investigated in EV-derived miRNA screening panels (Cho et al., 2019b). For example, miR-592 can target the prostaglandin D2 receptor (PTGDR) and the neutrophin receptor (NTR) p75, thus preventing proapoptotic signaling in neurons (Irmady et al., 2014; Sun et al., 2018). Furthermore, EV-derived miR-133b can inhibit connective tissue growth factor (CTGF) and Ras homology family member A (RhoA) expression allowing for neurite outgrowth (Xin et al., 2012, 2013b), whereas EV encapsulated miR-124 induces neurogenesis (Yang et al., 2017).

Thirdly is the elegant study by Han et al. (2020) who identified miR-410 encapsulated within umbilical cord-derived MSC-EVs as a potential mediator of therapeutic effects using a combination of *in-vitro*, molecular biology, *in-silico*, and *in-vivo* techniques. Specifically, they identified that miR-410 encapsulated MSC-EVs could inhibit neuronal apoptosis in OGD primary murine neuronal cells (Han et al., 2020). They also demonstrated, using the neonatal mouse, that EV-derived miR-410 reduced the neurological severity score, edema, and cerebral infarction volume compared to untreated HI animals (Han et al., 2020). Moreover, Han et al. (2020) demonstrated using both *in-silico* investigation and RNA expression that miR-410 may mediate its effects by down-regulating the expression of histone deactelayse 1 (HDAC1), which in turn enhances the expression of early growth response 2 (EGR2)/B-cell lymphoma (Bcl2) and prevents apoptosis.

Finally the fourth study, conducted by Du et al. (2021), demonstrated astrocyte-EV encapsulated miR-17-5p cargo reduced neuronal apoptosis and inflammation in the HI neonatal rat. Their study demonstrated an inverse relationship exists between miR-17-5p carried by astrocyte EVs and expression of the proapoptotic protein BCL2 Interacting Protein 2 (BNIP2) in the neonatal rat brain. Specifically, treatment using astrocyte-EV encapsulated miR-17-5p cargo resulted in a decrease in BNIP2 expression in the rat HI brain, while depletion of miR-17-5p cargo from astrocyte-EVs resulted in an increase in BNIP2 expression in the rat HI brain. Interestingly, an inverse relationship also existed between miR-17-5p and the proapoptotic protein BCL2 associated X, apoptosis regulator (BAX) (Wang et al., 2020). The authors also noted a decrease in BAX in the brains of rats treated with astrocyte-EVs. Hence, it is likely that the miR-17-5p cargo of astrocyte-EVs down-regulates both BAX and BNIP resulting in an increase in anti-apoptotic Bcl2 expression, which potentially contributes in part to the neuroprotective effects observed in this study.

It is possible that other molecular mediators that can confer neuroprotective and neuroregenerative properties are packaged as EV cargo. However, future work is required to identify these molecules, their mechanisms of action, whether they are cell of origin specific, and whether they preferentially target specific cell types to exert their effects.

### Reported Complications and Side-Effects

The results of the current literature are promising in terms of the therapeutic use of EVs in preclinical animal models of perinatal brain injury. Despite this, the administration of any therapeutic agent can bring with it complications and side effects. The administration of EVs appears well tolerated in the rat model and deleterious side effects, such as weight loss, death or clinical illness have not been reported (Drommelschmidt et al., 2017). Moreover, EV treatment of rats with perinatal brain injury results in an 18% increase in survival rate compared to those not treated (Thomi et al., 2019b). It is unclear whether this is true for the mouse model.

All the studies identified in this review investigated the role of EVs in the treatment of perinatal brain injury. In doing so, the researchers tend to focus on the brain alone. Yet it is possible that therapeutic administration of EVs likely results in the biodistribution of EVs to non-targeted regions. Presently, only three perinatal studies have sought to investigate the *in-vivo* biodistribution of EVs after administration and their off-target outcomes (Thomi et al., 2019b; Chu et al., 2020; Xin et al., 2020).

Experiments conducted in a P2 neonatal rat model of LPS/HI brain injury revealed that infrared dye (IRDye)^®^800CW-labeled MSC-EVs appeared in the frontal region of the brain, including the olfactory bulb, 30 min after intranasal administration and were evenly distributed throughout the brain within 3 h (Thomi et al., 2019b). Within non-targeted regions of the body only a small portion of EVs were identified in the trachea and gastrointestinal tract at 30 min and 3 h following intranasal administration and were absent in the spleen (Thomi et al., 2019b). No adverse effects were found in relation to the observed peripheral distribution. However, a limitation of this study is that it did not provide an extensive biodistribution investigation of organs well known to accumulate EVs, such as the liver, lung, kidney and heart (Wiklander et al., 2015). Nevertheless, this study strongly supports an intranasal route of administration as a means of efficiently delivering EVs to the developing brain. In addition, Chu et al. (2020) administered DiD-labeled EVs IC into a mouse model of term HI and detected their presence within the brain from 2 h post injection. Data from the same group using PKH67-labeled EVs delivered IC, established targeting to neurons (36.9%), astrocytes (21.5%), and microglia (34.3%) in the ipsilateral cortex (Xin et al., 2020). Unsurprisingly, given the method of EV delivery, PKH67-labeled EVs were found in other organs, including the liver, kidney and spleen (Chu et al., 2020; Xin et al., 2020). That both reports indicate that MSC-EVs can target the brain tissue and that distribution to the spleen is either absent or greatly reduced, suggests therapeutically administered EVs may not incite an immunological reaction. Regardless, the biodistribution of therapeutically delivered EVs requires further investigation as the research in this area is minimal. Understanding the *in-vivo* fate of EVs is of utmost importance for future therapeutic applications.

As a whole, the findings suggest that from a therapeutic perspective MSC-EVs are well tolerated and are less likely to accumulate in peripheral organs when administered intranasally. Whether IV administration has similar outcomes of biodistribu- tion and tolerance remains unknown and requires investigation.

## Discussion

The studies identified in this unbiased review investigating the potential use of EVs as a therapeutic treatment for perinatal brain injury, report an overall improvement in neurological outcomes. The studies highlight the ability of EVs to modulate inflammatory responses associated with both preterm and term acquired brain injuries. Because of these beneficial properties, they are successful in improving white matter and gray matter microstructural integrity through decreasing both gliosis and pro-inflammatory cytokine production and improving cell survival through amelioration of apoptosis. Additionally, EV therapy improved cognitive deficits (e.g., memory function and exploratory activity) and enhanced the performance in motor coordination function. These results are comparable to that of adult experimental models of neurological brain injury, including traumatic brain injury and stroke, where EV administration imparts neuroprotective and neuroregenerative effects (Xin et al., 2013a; Doeppner et al., 2015; Zhang et al., 2015). This suggests that with further research, EVs have the potential to be a useful therapeutic option in the treatment of perinatal brain injury.

Preclinical translation of EVs, however, is confounded by contrary publications on the effects of EVs. Contradictory results are most likely attributable to differences in animal experimental models, EV cell source, dosage of EVs, and route and timing of EV administration. This makes comparisons between studies difficult and limits interpretation of results. Although EVs represent a promising next-generation treatment for perinatal brain injury, future progress is dependent on a number of considerations. For example, while only 1/13 studied utilized astrocyte-EVs, 12/13 of the studies identified in this review utilized MSC-EVs, derived from different sources; either from the umbilical cord, umbilical cord blood, Wharton’s jelly of the human umbilical cord, or from adult bone marrow.

EVs isolated from MSC cultures of Wharton’s jelly have the notable advantage of coming from the umbilical cord, an extra-embryonic tissue, which is normally discarded at birth and free of ethical donor concerns and relatively low in cost (Frausin et al., 2015; Donders et al., 2018). Furthermore, there is evidence to show that MSCs derived from extra/embryonic/fetal tissues are more superior than adult bone marrow as they have a greater differentiation and expansion potential (Can and Karahuseyinoglu, 2007; Batsali et al., 2013). In addition, the controversy regarding the cellular heterogeneity in MSC cultures has crossed from cell-based therapies into EV-based therapies, thus appropriate characterization of the cell source (i.e., the MSC) is critical for reproducibility of therapeutic data utilizing MSC-EVs (Mo et al., 2016; Robey, 2017).

An important consideration will be to determine if the functional effects of MSC-EVs are reproducible using EVs from different cell sources. In this regard, it will also be crucial to establish any potential differences in mechanisms mediating EV therapeutic effects, which may exist between EV cell sources and their various small and large EV subpopulations. This is relevant, since heterogeneous populations exist and current isolation methods are relatively inadequate in resolving them (Gould and Raposo, 2013; Smith et al., 2015; Taylor and Shah, 2015; Willms et al., 2016, 2018; Jeppesen et al., 2019; Burbidge et al., 2020). Moreover, such comparisons are clearly necessary when designing EV therapeutic strategies for perinatal brain injury, since the immune-suppressive potential and thus ability to ameliorate neuroinflammation, including the potential to promote neuroregeneration are of paramount importance.

Current evidence suggests EVs secreted by cells exhibit target selection and that the EV cellular source must be matched to requirements of the target application and that EVs adopt a similar homing pattern to their cell source (Marcus and Leonard, 2013; Wiklander et al., 2015). Thus, it is feasible that generation of neural stem cell-derived EVs would be more likely to exhibit the highest distribution to the developing brain and confer neuroprotective benefits. Indeed, a few studies analyzing the efficacy of neural stem cell-derived EVs have shown robust results in both adolescent and adult stroke models (Webb et al., 2018a, b; Zhang et al., 2020).

Aside from the original cell type from which EVs are isolated, the impact of cell culture parameters and microenvironment in the production of EVs, including the passage number, seeding density, cell confluence, and the frequency and time of EV collection should be taken into consideration. While considerable progress has been made in EV production methods, the passage number is of particular relevance since evidence suggests the neuroprotective effect of human umbilical cord Wharton’s jelly MSCs (and their paracrine activities) diminish with increasing passage number (Bonab et al., 2006; Izadpanah et al., 2008; Patel et al., 2017; Dabrowska et al., 2018). Furthermore, reduced cell seeding density is attributable to greater production of EVs (Lieberman and Glaser, 1981; Théry et al., 2002; Patel et al., 2017). The culture conditions (e.g., oxygen concentration, media composition, 2D vs. 3D cultures) in which the source cells are cultured should be considered as these may influence not only the yield, but also the function of EVs released into the culture media (Park et al., 2018; Patel et al., 2018; Guo et al., 2019). This is particularly true given the differences Ophelders and colleagues noted in terms of the anti-inflammatory properties conveyed by MSCs and MSC-EVs following HI-induction in preterm fetal sheep (Jellema et al., 2013b; Ophelders et al., 2016). The authors postulated that such differences perhaps relate to fundamental differences in environments (Ophelders et al., 2016). Intuitively, endogenous MSCs have an innate capacity to sense their surrounding conditions and thus tailor their secreted factors (e.g., either pro- or anti-inflammatory EV cargo or other factors) to prevailing conditions. However, in the situation of *in-vivo* EV administration, EV cargo load is predetermined by the cellular response of cells to the culture environment.

Unfortunately, the lack of standardization of protocols between research groups investigating the therapeutic role of EVs in the treatment of perinatal brain injury adds a level of complexity in understanding the biochemical mechanisms at play. If future studies aim to standardise the cells from which EVs are isolated, including the methods, dosage and timing of EV administration, it may be possible to use an -omics approach to identify the mechanisms by which stem cell-derived EVs exert their functional effects. Once these biochemical mechanisms are identified we may be able to employ methods to enrich for candidate molecules in EVs as has been done in the study by Chu et al. (2020) or to bioengineer EVs containing molecules of interest to help optimize therapeutic molecule delivery.

Given the research funding environment, it is not surprising that individual research groups have chosen to focus on determining whether individual proteins (Annexin A1 and BDNF) and miRNA (miR-21a-5p, miR-7b-5p, miR-410, and miR-17-5p) are potential mediators of the neuroregenerative and neuroprotective effects observed in *in-vivo* models of HI treated using EVs. However, since each study utilized different EV isolation methods, vesicle cargo likely reflects the specific subpopulation of EVs isolated (Thery et al., 2018). Moreover, of the four studies investigating the role of miRNA EV cargo as mediators of therapeutic benefit, three (Chu et al., 2020; Han et al., 2020; Du et al., 2021) utilized enzymatic/detergent treatment. It is unclear whether the miRNA identified in the remaining study (Xin et al., 2020) was encapsulated within isolated EVs, attached to their surface of EVs, or simply contaminants co-isolated with the EV subpopulation. To help limit such ambiguity in future studies, researchers should be guided by the parameters recommended in the ISEV position papers (Mateescu et al., 2017; Thery et al., 2018) prior to the commencement of their research. By doing so will ensure optimal study design that include additional controls and detailed description of both sample processing and analysis that would enhance the overall quality and reproducibility of research publications.

Nevertheless, understanding the impact of individual mediators is critical to identifying which molecules that potentially mediate optimal neuroregenerative and neuroprotective responses from a nanotherapeutics development perspective, since synthetically manufactured EVs loaded with a specific cargo could be developed as an “off the shelf” product. However, biologically it is more likely that a combination of molecules (DNA/RNA/small RNA/protein/lipid/other) working synergistically will provide the greatest beneficial effect.

*In-vivo* biodistribution of EVs is determined by the route of administration (Wiklander et al., 2015, 2019). The rat study conducted by Thomi et al. (2019b) clearly demonstrates that intranasal administration of EVs is effective in targeting the brain. This route of administration is relatively non-invasive and would be easily translatable to the clinic. However, further research is required regarding the effectiveness of intranasal delivery of EVs in humans compared to animal models, especially given that the nasal olfactory mucosa (entry point to the brain) in rats covers approximately 45% more of the total nasal epithelium than in humans (Gross et al., 1982; Jiang et al., 2011).

In order to advance translation of EVs to the neonatal intensive care unit, appropriate preclinical animal models of brain injury are required to improve understanding of the underlying mechanism(s) by which EVs may exert protective effects. Such models will be crucial in establishing optimal routes and timing of administration and therapeutic effects, including any adverse effects. Furthermore, there must be appropriate negative control experiments. Surprisingly, of the perinatal studies reviewed, only two were identified where control experiments were performed using EV-depleted conditioned media (therapeutically inert EV control, isolated under identical conditions as experimental EVs) to exclude the possibility of effects through co-isolated proteins and common EV components (Shu et al., 2018; Han et al., 2020). Others utilized control EVs in the form of fibroblast-derived EVs (Ahn et al., 2021) and platelet-derived EVs (Kaminski et al., 2020), to establish whether neuroprotective and neuroregenerative effects were attributable to a cell of origin specific role of MSC-EVs, rather than EVs in general. Finally, the use of modified EVs either enriched for or depleted of cargo suspected to mediate observed beneficial effects (Chu et al., 2020; Xin et al., 2020; Du et al., 2021) has enabled determination of potential molecular mechanisms that could be exploited in future drug and nanotechnology developments aimed at treating perinatal brain injury. Notably, the addition of more than one control groups in *in-vivo* studies utilizing EVs as a potential therapeutic strategy for perinatal brain injury has increased since 2018 following publication of the MISEV guidelines, which advocates use of more control groups (Thery et al., 2018).

In relation to appropriate animal models, there are obvious anatomical and physiological differences between species and humans. The use of rodents and the extrapolation of data derived from these models has to be carefully considered since rodents have a lissencephalic (smooth surfaced) brain, with limited white matter tracts (Cook and Tymianski, 2011; Cai and Wang, 2016), that does not resemble the highly gyrencephalic human brain, and which makes interpretation of findings difficult. The fetal sheep model offers considerable advantages to bridge the gap between pre-clinical findings and clinical implementation. Specifically, the chronically instrumented preterm fetal sheep umbilical cord occlusion model closely replicates the common pathophysiological features of human preterm HI, namely diffuse white matter injury, with hippocampal as well as cortical and subcortical neuronal injury, thus emphasizing its translational character (Wassink et al., 2017; Cho et al., 2019a). Secondly, the translational nature of the fetal sheep model is best highlighted by preclinical studies of the near-term *in-utero* fetal sheep model (Gunn et al., 2005). These studies have provided key evidence of the safety and efficacy of hypothermia for treatment of HI encephalopathy, which is now the only viable therapy for HI encephalopathy in near-term or term infants. However, disadvantages of using such a large animal model include high cost of animal purchase, breeding, surgery, and housing. Consequently, it may not be possible to employ large numbers of animals into trials to demonstrate reproducibility; a factor, which researchers must consider in the pursuit of obtaining meaningful results when generating *in-vivo* data.

As the therapeutic application of EVs in the treatment of perinatal brain injury is in its infancy, there still are no studies reported which have tested the benefits of EVs in combination with hypothermia or other therapeutics. It is possible that administration of EVs will work synergistically with established therapeutic interventions and enhance clinical outcomes. Consequently, further work should be done to explore the potential of combination therapies.

Finally, although it is beyond the scope of this review, a unifying goal of recent and future advances in the field of EVs is to improve the therapeutic potential of EVs. More so, it is the need to find an effective way to treat newborns with brain injuries, which is one of the greatest challenges facing perinatal medicine. Achieving a promising outcome is most likely dependent on the composition of the EV cargo. As outlined previously, cargo found within EVs can include nucleic acids [miRNA (Valadi et al., 2007; Hunter et al., 2008), lncRNA (Gezer et al., 2014; Abramowicz and Story, 2020; Born et al., 2020), mRNA (Valadi et al., 2007; Kalra et al., 2012)], proteins (Simpson et al., 2008; Kalra et al., 2012), and DNA (Cai et al., 2013), and even cell surface receptors, cytosolic and nuclear proteins, and enzymes from the EV cell of origin (Andaloussi et al., 2013; Antonyak and Cerione, 2014; Yoon et al., 2014). Little is known regarding how the composition of cargo is specifically regulated by a particular cell. In all likelihood, there exists specific intracellular mechanisms that regulate the composition (Margolis and Sadovsky, 2019). However, artificial technologies could be implemented to achieve this in several ways; either by way of loading therapeutic cargo (including nucleic acids and small molecules compounds) and/or engineering EVs to display targeting moieties or protein therapeutics simultaneously on their membrane surface (Kooijmans et al., 2016; Antes et al., 2018; Murphy et al., 2019; Gupta et al., 2020). While there are no studies performed in perinatal animal models of brain injury, this approach clearly has the potential to offer considerable advantages around individualization of therapies that will improve neonatal care in the future.

## Conclusion

In recent years, there have been substantial efforts made to understand the characteristics of EVs and in particular, the therapeutic potential of those derived from stem cells. This has heralded an awareness that EVs and their cargo can become potential therapeutics in the treatment of brain disorders. However, as evidenced by this review, only a limited number of studies have sought to investigate their capacity to ameliorate the sequelae of perinatal brain injury. Therefore, definitive conclusions cannot be made regarding the use of EVs as a treatment for perinatal brain injury. Nevertheless, the available data demonstrates that therapeutic administration of EVs represents a promising new tool for the treatment of perinatal brain injury with studies utilizing *in-vitro* and *in-vivo* models suggesting that EVs will not only prevent, but also repair brain damage. Collectively, these studies highlight an enormous potential for future EV therapies, which requires further investigation. However, to ensure the validity of results and to advance the field of perinatal EV research, investigators should standardize future research practice by ensuring compliance to current minimal requirements for EV studies (MISEV guidelines) (Thery et al., 2018) and those of preclinical biological medicinal products (Lener et al., 2015). Equally important is the need for more systematic *in-vivo* studies, regarding the safety and potency of EVs, and the development of large-scale production, which is imperative to enable clinical translation.

## Author Contributions

TG conducted the unbiased literature search. TG and MF contributed equally to the preparation and writing of the review article. The final submitted article has been revised and approved by both authors.

## Conflict of Interest

The authors declare that the research was conducted in the absence of any commercial or financial relationships that could be construed as a potential conflict of interest.

## Publisher’s Note

All claims expressed in this article are solely those of the authors and do not necessarily represent those of their affiliated organizations, or those of the publisher, the editors and the reviewers. Any product that may be evaluated in this article, or claim that may be made by its manufacturer, is not guaranteed or endorsed by the publisher.

## Footnotes

1
ClinicalTrials.gov

## Abbreviations

ABCB1: ATP binding cassette subfamily member 1
AEA: Arachidonoylethanolamide
Akt: RAC-alpha serine/threonine-protein kinase
ALIX: ALG-2-interacting protein X
ANXA5: Annexin A5
ASC: apoptosis-associated speck-like protein containing a CARD
ATP: adenosine triphosphate
BAX: Bcl-2-associated X protein
BBB: blood-brain barrier
Bcl-XL: B-cell lymphoma extra-large
Bcl-2: B-cell lymphoma-2
BNIP2: BCL2 interacting protein 2
BDNF: brain derived neurotropic factor
CAT: catalase
CSF: cerebrospinal fluid
CNPase: 2 ′, 3,-cyclic nucleotide 3 ′ -phosphodiesterase
CNS: central nervous system
COX: cyclooxygenase
CP: cerebral palsy
CREB: cAMP response element-binding protein
CTGF: connective tissue growth factor
CXCL10: chemokine (C-X-C motif) ligand-10
DAMP: damage associated molecular pattern
DT-MRI: diffusion tensor magnetic resonance imaging
ECG: electrocardiogram
EEG: electroencephalogram
EGR2: early growth response 2
EpCam: epithelial cell adhesion molecule
Erk1/2: extracellular signal-regulated kinases
GA: gestational age
GABA: gamma-aminobutyric acid
GAPDH: glyceraldehyde 3-phosphate dehydrogenase
GPX: glutathione peroxidase
HDAC1: histone deactelayse 1
HI: hypoxia-ischemia
HIE: hypoxic-ischemic encephalopathy
hiPSC-nEVs: human induced pluripotent stem cell-derived EVs
HSF1: type 1 heat-shock factor
Iba1: ionized calcium binding adaptor molecule 1
IC: intracardial
ICAM: intercellular adhesion molecules
ICV: intracerebroventricular
IL: interleukin
iNOS: inducible nitric oxide synthase
IRDye: infrared dye
IV: intravenous
IVH: intraventricular hemorrhage
JAM1: junctional adhesion molecule-1
LPS: lipopolysaccharide
MAP2: microtubule-associated protein 2
MBP: myelin basic protein
MDA: malondialdehyde
miRNA: microRNA
MISEV: minimal information for studies of extracellular vesicles 2018
MMP-9: matrix metallopeptidase-9; Matrix metallopeptidases (MMPs)
MOG: myelin oligodendroglia glycoprotein
MRI: magnetic resonance imaging
MSC: mesenchymal stem cell-derived extracellular vesicles
NF κβ: nuclear factor kappa beta
NRLP1: nod-like receptor protein 1
NTR: neutrophin receptor
OGD: oxygen-glucose deprivation
P: postnatal day
P2RX7: purinergic receptor P2X7
PDGF-BB: platelet-derived growth factor-BB
PDGFR β: platelet-derived growth factor receptor beta
PEG: polyethylene glycol
PLP: myelin proteolipid protein
PRISMA: Preferred Reporting Items for Systematic Reviews and Meta-Analyses
PrP^C^: cellular prion protein
PTGDR: prostaglandin D2 receptor
PTX3: pentraxin 3
REST: restriction element-1 silencing transcription factor
RhoA: Ras homology family member A
STI1: stress-inducible protein 1
SEM: scanning electron microscopy
SOD: superoxide dismutase
SVZ: subventricular zone
TAG1: transiently expressed axonal surface glycoprotein-1
TEM: transmission electron microscopy
Timp3: tissue inhibitor of metalloproteinase 3
TkrB: tyrosine kinase receptor B
TNF: tumor necrosis factor
TRPS: tunable resistive pulse sensing
TSG101: tumor susceptibility gene 101
TTC: 2,3,5-tryphenyl tetrazolium chloride
TUNEL: terminal deoxynucleotidyl transferase dUTP nick end labeling.

## References

[B1] Abramowicz A. Story M. D. (2020). The long and short of it: the emerging roles of non-coding RNA in small extracellular vesicles. *Cancers (Basel)* 12:1445. 10.3390/cancers12061445 32498257 PMC7352322

[B2] Adamiak M. Cheng G. Bobis-Wozowicz S. Zhao L. Kedracka-Krok S. Samanta A. (2018). Induced pluripotent stem cell (iPSC)-derived extracellular vesicles are safer and more effective for cardiac repair than iPSCs. *Circ. Res.* 122 296–309. 10.1161/circresaha.117.311769 29118058 PMC5775034

[B3] Ahn S. Y. Chang Y. S. Sung D. K. Sung S. I. Ahn J. Y. Park W. S. (2017). Pivotal role of brain-derived neurotrophic factor secreted by mesenchymal stem cells in severe intraventricular hemorrhage in newborn rats. *Cell Transplant.* 26 145–156. 10.3727/096368916x692861 27535166 PMC5657690

[B4] Ahn S. Y. Sung D. K. Kim Y. E. Sung S. Chang Y. S. Park W. S. (2021). Brain-derived neurotropic factor mediates neuroprotection of mesenchymal stem cell-derived extracellular vesicles against severe intraventricular hemorrhage in newborn rats. *Stem Cells Trans. Med.* 10 374–384. 10.1002/sctm.20-0301 33319929 PMC7900593

[B5] Ajmo C. T. Jr. Vernon D. O. Collier L. Hall A. A. Garbuzova-Davis S. Willing A. (2008). The spleen contributes to stroke-induced neurodegeneration. *J. Neurosci. Res.* 86 2227–2234. 10.1002/jnr.21661 18381759 PMC2680137

[B6] Albertsson A. M. Bi D. Duan L. Zhang X. Leavenworth J. W. Qiao L. (2014). The immune response after hypoxia-ischemia in a mouse model of preterm brain injury. *J. Neuroinflammation* 11:153. 10.1186/s12974-014-0153-z PMC4172879 25187205

[B7] Alexandrou G. Mårtensson G. Skiöld B. Blennow M. Adén U. Vollmer B. (2014). White matter microstructure is influenced by extremely preterm birth and neonatal respiratory factors. *Acta Paediatr.* 103 48–56. 24118089 10.1111/apa.12445

[B8] Ananth C. V. Joseph K. S. Oyelese Y. Demissie K. Vintzileos A. M. (2005). Trends in preterm birth and perinatal mortality among singletons: United States, 1989 through 2000. *Obstet. Gynecol.* 105 1084–1091. 10.1097/01.aog.0000158124.96300.c7 15863548

[B9] Andaloussi S. E. L. Mäger I. Breakefield X. O. Wood M. J. (2013). Extracellular vesicles: biology and emerging therapeutic opportunities. *Nat. Rev. Drug Discov.* 12 347–357. 10.1038/nrd3978 23584393

[B10] Antes T. J. Middleton R. C. Luther K. M. Ijichi T. Peck K. A. Liu W. J. (2018). Targeting extracellular vesicles to injured tissue using membrane cloaking and surface display. *J. Nanobiotechnol.* 16:61. 10.1186/s12951-018-0388-4 PMC6116387 30165851

[B11] Antonyak M. A. Cerione R. A. (2014). Microvesicles as mediators of intercellular communication in cancer. *Methods Mol. Biol.* 1165 147–173. 10.1007/978-1-4939-0856-1_11 24839024

[B12] Arantes R. M. Andrews N. W. (2006). A role for synaptotagmin VII-regulated exocytosis of lysosomes in neurite outgrowth from primary sympathetic neurons. *J. Neurosci.* 26 4630–4637. 10.1523/jneurosci.0009-06.2006 16641243 PMC6674075

[B13] Azzopardi D. V. Strohm B. Edwards A. D. Dyet L. Halliday H. L. Juszczak E. (2009). Moderate hypothermia to treat perinatal asphyxial encephalopathy. *N. Engl. J. Med.* 361 1349–1358. 19797281 10.1056/NEJMoa0900854

[B14] Azzopardi D. Strohm B. Marlow N. Brocklehurst P. Deierl A. Eddama O. (2014). Effects of hypothermia for perinatal asphyxia on childhood outcomes. *N. Engl. J. Med.* 371 140–149. 25006720 10.1056/NEJMoa1315788

[B15] Baburamani A. A. Supramaniam V. G. Hagberg H. Mallard C. (2014). Microglia toxicity in preterm brain injury. *Reprod. Toxicol.* 48 106–112. 10.1016/j.reprotox.2014.04.002 24768662 PMC4155935

[B16] Back S. A. (2014). Cerebral white and gray matter injury in newborns: new insights into pathophysiology and management. *Clin. Perinatol.* 41 1–24. 10.1016/j.clp.2013.11.001 24524444 PMC3947650

[B17] Back S. A. (2015). Brain injury in the preterm infant: new horizons for pathogenesis and prevention. *Pediatr. Neurol.* 53 185–192. 10.1016/j.pediatrneurol.2015.04.006 26302698 PMC4550810

[B18] Back S. A. Miller S. P. (2014). Brain injury in premature neonates: a primary cerebral dysmaturation disorder? *Ann. Neurol.* 75 469–486. 24615937 10.1002/ana.24132 PMC5989572

[B19] Back S. A. Luo N. L. Borenstein N. S. Levine J. M. Volpe J. J. Kinney H. C. (2001). Late oligodendrocyte progenitors coincide with the developmental window of vulnerability for human perinatal white matter injury. *J. Neurosci.* 21 1302–1312. 10.1523/jneurosci.21-04-01302.2001 11160401 PMC6762224

[B20] Bahrini I. Song J.-H. Diez D. Hanayama R. (2015). Neuronal exosomes facilitate synaptic pruning by up-regulating complement factors in microglia. *Sci. Rep.* 5:7989. 10.1038/srep07989 PMC4303875 25612542

[B21] Bakhti M. Winter C. Simons M. (2011). Inhibition of myelin membrane sheath formation by oligodendrocyte-derived exosome-like vesicles. *J. Biol. Chem.* 286 787–796. 10.1074/jbc.m110.190009 20978131 PMC3013037

[B22] Ballabh P. Hu F. Kumarasiri M. Braun A. Nedergaard M. (2005). Development of tight junction molecules in blood vessels of germinal matrix, cerebral cortex, and white matter. *Pediatr. Res.* 58 791–798. 10.1203/01.pdr.0000180535.14093.fb 16189211

[B23] Banigan M. G. Kao P. F. Kozubek J. A. Winslow A. R. Medina J. Costa J. (2013). Differential expression of exosomal microRNAs in prefrontal cortices of schizophrenia and bipolar disorder patients. *PLoS One* 8:e48814. 10.1371/journal.pone.0048814 23382797 PMC3559697

[B24] Baraniak P. R. McDevitt T. C. (2010). Stem cell paracrine actions and tissue regeneration. *Regen. Med.* 5 121–143. 10.2217/rme.09.74 20017699 PMC2833273

[B25] Bassler D. Stoll B. J. Schmidt B. Asztalos E. V. Roberts R. S. Robertson C. M. (2009). Using a count of neonatal morbidities to predict poor outcome in extremely low birth weight infants: added role of neonatal infection. *Pediatrics* 123 313–318. 10.1542/peds.2008-0377 19117897 PMC2829863

[B26] Batsali A. K. Kastrinaki M. C. Papadaki H. A. Pontikoglou C. (2013). Mesenchymal stem cells derived from Wharton’s Jelly of the umbilical cord: biological properties and emerging clinical applications. *Curr. Stem Cell Res. Ther.* 8 144–155. 10.2174/1574888x11308020005 23279098

[B27] Beck S. Wojdyla D. Say L. Betran A. P. Merialdi M. Requejo J. H. (2010). The worldwide incidence of preterm birth: a systematic review of maternal mortality and morbidity. *Bull. World Health Organ.* 88 31–38. 10.2471/blt.08.062554 20428351 PMC2802437

[B28] Behrman R. E. Butler A. S. (2007). *Preterm Birth : Causes, Consequences, and Prevention.* Washington, DC: National Academies Press (US). 20669423

[B29] Bennet L. Roelfsema V. George S. Dean J. M. Emerald B. S. Gunn A. J. (2007). The effect of cerebral hypothermia on white and grey matter injury induced by severe hypoxia in preterm fetal sheep. *J. Physiol.* 578 491–506. 10.1113/jphysiol.2006.119602 17095565 PMC2075155

[B30] Bianco F. Perrotta C. Novellino L. Francolini M. Riganti L. Menna E. (2009). Acid sphingomyelinase activity triggers microparticle release from glial cells. *EMBO J.* 28 1043–1054. 10.1038/emboj.2009.45 19300439 PMC2664656

[B31] Bianco F. Pravettoni E. Colombo A. Schenk U. Möller T. Matteoli M. (2005). Astrocyte-derived ATP induces vesicle shedding and IL-1 beta release from microglia. *J. Immunol.* 174 7268–7277. 10.4049/jimmunol.174.11.7268 15905573

[B32] Bifrare Y.-D. Kummer J. Joss P. Täuber M. G. Leib S. L. (2005). Brain-Derived neurotrophic factor protects against multiple forms of brain injury in bacterial meningitis. *J. Infect. Dis.* 191 40–45. 10.1086/426399 15593001

[B33] Blencowe H. Cousens S. Chou D. Oestergaard M. Say L. Moller A. B. (2013a). Born too soon: the global epidemiology of 15 million preterm births. *Reprod. Health* 10:S2. 10.1186/1742-4755-10-S1-S2 PMC3828585 24625129

[B34] Blencowe H. Cousens S. Oestergaard M. Z. Chou D. Moller A. B. Narwal R. (2012). National, regional, and worldwide estimates of preterm birth rates in the year 2010 with time trends since 1990 for selected countries: a systematic analysis and implications. *Lancet* 379 2162–2172. 10.1016/s0140-6736(12)60820-4 22682464

[B35] Blencowe H. Lee A. C. Cousens S. Bahalim A. Narwal R. Zhong N. (2013b). Preterm birth-associated neurodevelopmental impairment estimates at regional and global levels for 2010. *Pediatr. Res.* 74(Suppl. 1) 17–34. 10.1038/pr.2013.204 24366461 PMC3873710

[B36] Bonab M. M. Alimoghaddam K. Talebian F. Ghaffari S. H. Ghavamzadeh A. Nikbin B. (2006). Aging of mesenchymal stem cell in vitro. *BMC Cell Biol.* 7:14. 10.1186/1471-2121-7-14 16529651 PMC1435883

[B37] Bonafede R. Scambi I. Peroni D. Potrich V. Boschi F. Benati D. (2016). Exosome derived from murine adipose-derived stromal cells: neuroprotective effect on in vitro model of amyotrophic lateral sclerosis. *Exp. Cell Res.* 340 150–158. 10.1016/j.yexcr.2015.12.009 26708289

[B38] Born L. J. Harmon J. W. Jay S. M. (2020). Therapeutic potential of extracellular vesicle−associated long noncoding RNA. *Bioeng. Transl. Med.* 5:e10172. 10.1002/btm2.10172 PMC7510462 33005738

[B39] Briatore E. Ferrari F. Pomero G. Boghi A. Gozzoli L. Micciolo R. (2013). EEG findings in cooled asphyxiated newborns and correlation with site and severity of brain damage. *Brain Dev.* 35 420–426. 10.1016/j.braindev.2012.07.002 22871392

[B40] Budnik V. Ruiz-Canada C. Wendler F. (2016). Extracellular vesicles round off communication in the nervous system. *Nat. Rev. Neurosci.* 17 160–172. 10.1038/nrn.2015.29 26891626 PMC4989863

[B41] Burbidge K. Zwikelmaier V. Cook B. Long M. M. Balva B. Lonigro M. (2020). Cargo and cell-specific differences in extracellular vesicle populations identified by multiplexed immunofluorescent analysis. *J. Extracell. Vesicles* 9:1789326. 10.1080/20013078.2020.1789326 32944176 PMC7480458

[B42] Burda J. E. Sofroniew M. V. (2014). Reactive gliosis and the multicellular response to CNS damage and disease. *Neuron* 81 229–248. 24462092 10.1016/j.neuron.2013.12.034 PMC3984950

[B43] Buser J. R. Maire J. Riddle A. Gong X. Nguyen T. Nelson K. (2012). Arrested preoligodendrocyte maturation contributes to myelination failure in premature infants. *Ann. Neurol.* 71 93–109. 10.1002/ana.22627 22275256 PMC3270934

[B44] Cai B. Wang N. (2016). Large animal stroke models vs. rodent stroke models, pros and cons, and combination? *Acta Neurochir. Suppl.* 121 77–81. 10.1007/978-3-319-18497-5_13 26463926

[B45] Cai J. Han Y. Ren H. Chen C. He D. Zhou L. (2013). Extracellular vesicle-mediated transfer of donor genomic DNA to recipient cells is a novel mechanism for genetic influence between cells. *J. Mol. Cell Biol.* 5 227–238. 10.1093/jmcb/mjt011 23580760 PMC3733418

[B46] Cai J. Wu J. Wang J. Li Y. Hu X. Luo S. (2020). Extracellular vesicles derived from different sources of mesenchymal stem cells: therapeutic effects and translational potential. *Cell Biosci.* 10:69. 10.1186/s13578-020-00427-x PMC7245623 32483483

[B47] Caldú X. Narberhaus A. Junqué C. Giménez M. Vendrell P. Bargalló N. (2006). Corpus callosum size and neuropsychologic impairment in adolescents who were born preterm. *J. Child Neurol.* 21 406–410. 10.1177/08830738060210050801 16901446

[B48] Campanella C. Caruso Bavisotto C. Logozzi M. Marino Gammazza A. Mizzoni D. Cappello F. (2019). On the choice of the extracellular vesicles for therapeutic purposes. *Int. J. Mol. Sci.* 20:236. 10.3390/ijms20020236 30634425 PMC6359369

[B49] Can A. Karahuseyinoglu S. (2007). Concise review: human umbilical cord stroma with regard to the source of fetus-derived stem cells. *Stem Cells* 25 2886–2895. 10.1634/stemcells.2007-0417 17690177

[B50] Chen A. Xiong L. J. Tong Y. Mao M. (2013). The neuroprotective roles of BDNF in hypoxic ischemic brain injury. *Biomed. Rep.* 1 167–176. 10.3892/br.2012.48 24648914 PMC3956206

[B51] Chen C. C. Liu L. Ma F. Wong C. W. Guo X. E. Chacko J. V. (2016). Elucidation of exosome migration across the blood-brain barrier model in vitro. *Cell. Mol. Bioeng.* 9 509–529. 10.1007/s12195-016-0458-3 28392840 PMC5382965

[B52] Chen S. Datta-Chaudhuri A. Deme P. Dickens A. Dastgheyb R. Bhargava P. (2019). Lipidomic characterization of extracellular vesicles in human serum. *J. Circ. Biomark.* 8:1849454419879848. 10.1177/1849454419879848 PMC6769212 31632506

[B53] Chen X. Threlkeld S. W. Cummings E. E. Juan I. Makeyev O. Besio W. G. (2012). Ischemia-reperfusion impairs blood-brain barrier function and alters tight junction protein expression in the ovine fetus. *Neuroscience* 226 89–100. 10.1016/j.neuroscience.2012.08.043 22986172 PMC3490041

[B54] Cho K. H. T. Wassink G. Galinsky R. Xu B. Mathai S. Dhillon S. K. (2019a). Protective effects of delayed intraventricular TLR7 agonist administration on cerebral white and gray matter following asphyxia in the preterm fetal sheep. *Sci. Rep.* 9:9562. 10.1038/s41598-019-45872-y PMC6606639 31267031

[B55] Cho K. H. T. Xu B. Blenkiron C. Fraser M. (2019b). Emerging roles of miRNAs in brain development and perinatal brain injury. *Front. Physiol.* 10:227. 10.3389/fphys.2019.00227 30984006 PMC6447777

[B56] Chu X. Liu D. Li T. Ke H. Xin D. Wang S. (2020). Hydrogen sulfide-modified extracellular vesicles from mesenchymal stem cells for treatment of hypoxic-ischemic brain injury. *J. Control. Release* 328 13–27. 10.1016/j.jconrel.2020.08.037 32858071

[B57] Cocucci E. Meldolesi J. (2015). Ectosomes and exosomes: shedding the confusion between extracellular vesicles. *Trends Cell Biol.* 25 364–372. 10.1016/j.tcb.2015.01.004 25683921

[B58] Coleman B. M. Hill A. F. (2015). Extracellular vesicles – Their role in the packaging and spread of misfolded proteins associated with neurodegenerative diseases. *Semin. Cell Dev. Biol.* 40 89–96. 10.1016/j.semcdb.2015.02.007 25704308

[B59] Cook D. J. Tymianski M. (2011). Translating promising preclinical neuroprotective therapies to human stroke trials. *Expert Rev. Cardiovasc. Ther.* 9 433–449. 10.1586/erc.11.34 21517728

[B60] Coolen M. Katz S. Bally-Cuif L. (2013). miR-9: a versatile regulator of neurogenesis. *Front. Cell. Neurosci.* 7:220. 10.3389/fncel.2013.00220 24312010 PMC3834235

[B61] Crowther C. A. Middleton P. F. Voysey M. Askie L. Duley L. Pryde P. G. (2017). Assessing the neuroprotective benefits for babies of antenatal magnesium sulphate: an individual participant data meta-analysis. *PLoS Med.* 14:e1002398. 10.1371/journal.pmed.1002398 28976987 PMC5627896

[B62] Dabrowska S. Sypecka J. Jablonska A. Strojek L. Wielgos M. Domanska-Janik K. (2018). Neuroprotective potential and paracrine activity of stromal Vs. culture-expanded hMSC derived from wharton jelly under co-cultured with hippocampal organotypic slices. *Mol. Neurobiol.* 55 6021–6036. 10.1007/s12035-017-0802-1 29134515 PMC5994221

[B63] Danzer K. M. Kranich L. R. Ruf W. P. Cagsal-Getkin O. Winslow A. R. Zhu L. (2012). Exosomal cell-to-cell transmission of alpha synuclein oligomers. *Mol. Neurodegener.* 7:42. 10.1186/1750-1326-7-42 22920859 PMC3483256

[B64] de Almeida P. E. Ransohoff J. D. Nahid A. Wu J. C. (2013). Immunogenicity of pluripotent stem cells and their derivatives. *Circ. Res.* 112 549–561. 10.1161/circresaha.111.249243 23371903 PMC3638957

[B65] de Haan T. R. Bijleveld Y. A. Van Der Lee J. H. Groenendaal F. Van Den Broek M. P. Rademaker C. M. (2012). Pharmacokinetics and pharmacodynamics of medication in asphyxiated newborns during controlled hypothermia. The PharmaCool multicenter study. *BMC Pediatr.* 12:45. 10.1186/1471-2431-12-45 22515424 PMC3358232

[B66] de Paula S. Greggio S. Dacosta J. C. (2010). Use of stem cells in perinatal asphyxia: from bench to bedside. *J. Pediatr. (Rio J)* 86 451–464. 10.2223/jped.2035 21140037

[B67] de Rivero Vaccari J. P. Brand F. III Adamczak S. Lee S. W. Perez-Barcena J. Wang M. Y. (2016). Exosome-mediated inflammasome signaling after central nervous system injury. *J. Neurochem.* 136(Suppl. 1) 39–48. 10.1111/jnc.13036 25628216 PMC4516699

[B68] Deng M. Xiao H. Peng H. Yuan H. Xu Y. Zhang G. (2018). Preservation of neuronal functions by exosomes derived from different human neural cell types under ischemic conditions. *Eur. J. Neurosci.* 47 150–157. 10.1111/ejn.13784 29178548

[B69] Deng Y. Y. Lu J. Ling E. A. Kaur C. (2010). Microglia-derived macrophage colony stimulating factor promotes generation of proinflammatory cytokines by astrocytes in the periventricular white matter in the hypoxic neonatal brain. *Brain Pathol.* 20 909–925. 20406232 10.1111/j.1750-3639.2010.00387.x PMC8094857

[B70] Dharap A. Bowen K. Place R. Li L. C. Vemuganti R. (2009). Transient focal ischemia induces extensive temporal changes in rat cerebral microRNAome. *J. Cereb. Blood Flow Metab.* 29 675–687. 10.1038/jcbfm.2008.157 19142192 PMC2743462

[B71] Dhillon S. K. Gunn A. J. Jung Y. Mathai S. Bennet L. Fraser M. (2015). Lipopolysaccharide-Induced preconditioning attenuates apoptosis and differentially regulates TLR4 and TLR7 gene expression after ischemia in the preterm ovine fetal brain. *Dev. Neurosci.* 37 497–514. 10.1159/000433422 26184807

[B72] Dickens A. M. Tovar Y. R. L. B. Yoo S. W. Trout A. L. Bae M. Kanmogne M. (2017). Astrocyte-shed extracellular vesicles regulate the peripheral leukocyte response to inflammatory brain lesions. *Sci. Signal.* 10:eaai7696. 10.1126/scisignal.aai7696 28377412 PMC5590230

[B73] Doeppner T. R. Herz J. Gorgens A. Schlechter J. Ludwig A. K. Radtke S. (2015). Extracellular vesicles improve post-stroke neuroregeneration and prevent postischemic immunosuppression. *Stem Cells Transl. Med.* 4 1131–1143. 10.5966/sctm.2015-0078 26339036 PMC4572905

[B74] Dolcetti E. Bruno A. Guadalupi L. Rizzo F. R. Musella A. Gentile A. (2020). Emerging role of extracellular vesicles in the pathophysiology of multiple sclerosis. *Int. J. Mol. Sci.* 21:7336. 10.3390/ijms21197336 33020408 PMC7582271

[B75] Donders R. Bogie J. F. J. Ravanidis S. Gervois P. Vanheusden M. Marée R. (2018). Human Wharton’s jelly-derived stem cells display a distinct immunomodulatory and proregenerative transcriptional signature compared to bone marrow-derived stem cells. *Stem Cells Dev.* 27 65–84. 10.1089/scd.2017.0029 29267140

[B76] Donega V. Nijboer C. H. Van Tilborg G. Dijkhuizen R. M. Kavelaars A. Heijnen C. J. (2014). Intranasally administered mesenchymal stem cells promote a regenerative niche for repair of neonatal ischemic brain injury. *Exp. Neurol.* 261 53–64. 10.1016/j.expneurol.2014.06.009 24945601

[B77] Drago F. Lombardi M. Prada I. Gabrielli M. Joshi P. Cojoc D. (2017). ATP modifies the proteome of extracellular vesicles released by microglia and influences their action on astrocytes. *Front. Pharmacol.* 8:910. 10.3389/fphar.2017.00910 29321741 PMC5733563

[B78] Drommelschmidt K. Serdar M. Bendix I. Herz J. Bertling F. Prager S. (2017). Mesenchymal stem cell-derived extracellular vesicles ameliorate inflammation-induced preterm brain injury. *Brain Behav. Immun.* 60 220–232. 10.1016/j.bbi.2016.11.011 27847282

[B79] Du L. Jiang Y. Sun Y. (2021). Astrocyte-derived exosomes carry microRNA-17-5p to protect neonatal rats from hypoxic-ischemic brain damage via inhibiting BNIP-2 expression. *NeuroToxicology* 83 28–39. 10.1016/j.neuro.2020.12.006 33309839

[B80] Emmanouilidou E. Melachroinou K. Roumeliotis T. Garbis S. D. Ntzouni M. Margaritis L. H. (2010). Cell-produced alpha-synuclein is secreted in a calcium-dependent manner by exosomes and impacts neuronal survival. *J. Neurosci.* 30 6838–6851. 10.1523/jneurosci.5699-09.2010 20484626 PMC3842464

[B81] Engelhardt E. Inder T. E. Alexopoulos D. Dierker D. L. Hill J. Van Essen D. (2015). Regional impairments of cortical folding in premature infants. *Ann. Neurol.* 77 154–162. 10.1002/ana.24313 25425403 PMC4324979

[B82] Feliciano D. M. Zhang S. Nasrallah C. M. Lisgo S. N. Bordey A. (2014). Embryonic cerebrospinal fluid nanovesicles carry evolutionarily conserved molecules and promote neural stem cell amplification. *PLoS One* 9:e88810. 10.1371/journal.pone.0088810 24533152 PMC3923048

[B83] Fevrier B. Vilette D. Archer F. Loew D. Faigle W. Vidal M. (2004). Cells release prions in association with exosomes. *Proc. Natl. Acad. Sci. U.S.A.* 101 9683–9688. 10.1073/pnas.0308413101 15210972 PMC470735

[B84] Fiandaca M. S. Kapogiannis D. Mapstone M. Boxer A. Eitan E. Schwartz J. B. (2015). Identification of preclinical Alzheimer’s disease by a profile of pathogenic proteins in neurally derived blood exosomes: a case-control study. *Alzheimers Dement.* 11 600–607.e601. 25130657 10.1016/j.jalz.2014.06.008 PMC4329112

[B85] Fischer U. M. Harting M. T. Jimenez F. Monzon-Posadas W. O. Xue H. Savitz S. I. (2009). Pulmonary passage is a major obstacle for intravenous stem cell delivery: the pulmonary first-pass effect. *Stem Cells Dev.* 18 683–692. 10.1089/scd.2008.0253 19099374 PMC3190292

[B86] Fitzner D. Schnaars M. Van Rossum D. Krishnamoorthy G. Dibaj P. Bakhti M. (2011). Selective transfer of exosomes from oligodendrocytes to microglia by macropinocytosis. *J. Cell Sci.* 124 447–458. 10.1242/jcs.074088 21242314

[B87] Fraser M. Bennet L. Gunning M. Williams C. Gluckman P. D. George S. (2005). Cortical electroencephalogram suppression is associated with post-ischemic cortical injury in 0.65 gestation fetal sheep. *Brain Res. Dev. Brain Res.* 154 45–55. 10.1016/j.devbrainres.2004.10.002 15617754

[B88] Fraser M. Bennet L. Helliwell R. Wells S. Williams C. Gluckman P. (2007). Regional specificity of magnetic resonance imaging and histopathology following cerebral ischemia in preterm fetal sheep. *Reprod. Sci.* 14 182–191. 10.1177/1933719107299612 17636230

[B89] Fraser M. Bennet L. Van Zijl P. L. Mocatta T. J. Williams C. E. Gluckman P. D. (2008). Extracellular amino acids and lipid peroxidation products in periventricular white matter during and after cerebral ischemia in preterm fetal sheep. *J. Neurochem.* 105 2214–2223. 10.1111/j.1471-4159.2008.05313.x 18315562

[B90] Frausin S. Viventi S. Verga Falzacappa L. Quattromani M. J. Leanza G. Tommasini A. (2015). Wharton’s jelly derived mesenchymal stromal cells: biological properties, induction of neuronal phenotype and current applications in neurodegeneration research. *Acta Histochem.* 117 329–338. 25747736 10.1016/j.acthis.2015.02.005

[B91] Fröhlich D. Kuo W. P. Frühbeis C. Sun J. J. Zehendner C. M. Luhmann H. J. (2014). Multifaceted effects of oligodendroglial exosomes on neurons: impact on neuronal firing rate, signal transduction and gene regulation. *Philos. Trans. R. Soc. Lond. B Biol. Sci.* 369:20130510. 10.1098/rstb.2013.0510 25135971 PMC4142031

[B92] Frühbeis C. Fröhlich D. Kuo W. P. Amphornrat J. Thilemann S. Saab A. S. (2013a). Neurotransmitter-triggered transfer of exosomes mediates oligodendrocyte-neuron communication. *PLoS Biol.* 11:e1001604. 10.1371/journal.pbio.1001604 23874151 PMC3706306

[B93] Frühbeis C. Fröhlich D. Kuo W. P. Krämer-Albers E.-M. (2013b). Extracellular vesicles as mediators of neuron-glia communication. *Front. Cell. Neurosci.* 7:182. 10.3389/fncel.2013.00182 24194697 PMC3812991

[B94] Fu Y. Karbaat L. Wu L. Leijten J. Both S. K. Karperien M. (2017). Trophic effects of mesenchymal stem cells in tissue regeneration. *Tissue Eng. Part B Rev.* 23 515–528. 28490258 10.1089/ten.TEB.2016.0365

[B95] Galinsky R. Lear C. A. Dean J. M. Wassink G. Dhillon S. K. Fraser M. (2018). Complex interactions between hypoxia-ischemia and inflammation in preterm brain injury. *Dev. Med. Child Neurol.* 60 126–133. 10.1111/dmcn.13629 29194585

[B96] Gezer U. Özgür E. Cetinkaya M. Isin M. Dalay N. (2014). Long non-coding RNAs with low expression levels in cells are enriched in secreted exosomes. *Cell Biol. Int.* 38 1076–1079. 24798520 10.1002/cbin.10301

[B97] Gluckman P. D. Wyatt J. S. Azzopardi D. Ballard R. Edwards A. D. Ferriero D. M. (2005). Selective head cooling with mild systemic hypothermia after neonatal encephalopathy: multicentre randomised trial. *Lancet* 365 663–670. 10.1016/s0140-6736(05)70932-6 15721471

[B98] Goetzl L. Darbinian N. Goetzl E. J. (2016). Novel window on early human neurodevelopment via fetal exosomes in maternal blood. *Ann. Clin. Transl. Neurol.* 3 381–385. 10.1002/acn3.296 27231707 PMC4863750

[B99] Goetzl L. Darbinian N. Merabova N. (2019). Noninvasive assessment of fetal central nervous system insult: potential application to prenatal diagnosis. *Prenat. Diagn.* 39 609–615. 10.1002/pd.5474 31069822

[B100] Goetzl L. Merabova N. Darbinian N. Martirosyan D. Poletto E. Fugarolas K. (2017). Diagnostic potential of neural exosome cargo as biomarkers for acute brain injury. *Ann. Clin. Transl. Neurol.* 5 4–10. 10.1002/acn3.499 29376087 PMC5771318

[B101] Gomzikova M. O. James V. Rizvanov A. A. (2019). Therapeutic application of mesenchymal stem cells derived extracellular vesicles for immunomodulation. *Front. Immunol.* 10:2663. 10.3389/fimmu.2019.02663 31849929 PMC6889906

[B102] Gong J. Körner R. Gaitanos L. Klein R. (2016). Exosomes mediate cell contact–independent ephrin-Eph signaling during axon guidance. *J. Cell Biol.* 214 35–44. 10.1083/jcb.201601085 27354374 PMC4932373

[B103] Gopagondanahalli K. R. Li J. Fahey M. C. Hunt R. W. Jenkin G. Miller S. L. (2016). Preterm hypoxic-ischemic encephalopathy. *Front. Pediatr.* 4:114. 10.3389/fped.2016.00114 27812521 PMC5071348

[B104] Gould S. J. Raposo G. (2013). As we wait: coping with an imperfect nomenclature for extracellular vesicles. *J. Extracell. Vesicles* 2:20389. 10.3402/jev.v2i0.20389 24009890 PMC3760635

[B105] Gross E. A. Swenberg J. A. Fields S. Popp J. A. (1982). Comparative morphometry of the nasal cavity in rats and mice. *J. Anat.* 135 83–88. 7130058 PMC1168130

[B106] Gunn A. J. Battin M. Gluckman P. D. Gunn T. R. Bennet L. (2005). Therapeutic hypothermia: from lab to NICU. *J. Perinat. Med.* 33 340–346. 16207121 10.1515/JPM.2005.061

[B107] Guo Y. Tan J. Miao Y. Sun Z. Zhang Q. (2019). Effects of microvesicles on cell apoptosis under hypoxia. *Oxid. Med. Cell. Longev.* 2019:5972152. 10.1155/2019/5972152 PMC6501227 31178970

[B108] Gupta D. Wiklander O. P. B. Görgens A. Conceição M. Corso G. Liang X. (2020). Engineering of extracellular vesicles for display of protein biotherapeutics. *bioRxiv* [preprint]. 10.1101/2020.06.14.149823

[B109] Gussenhoven R. Klein L. Ophelders D. Habets D. H. J. Giebel B. Kramer B. W. (2019). Annexin A1 as neuroprotective determinant for blood-brain barrier integrity in neonatal hypoxic-ischemic encephalopathy. *J. Clin. Med.* 8:137. 10.3390/jcm8020137 30682787 PMC6406389

[B110] Hagberg H. Gilland E. Bona E. Hanson L. Å Hahn-Zoric M. Blennow M. (1996). Enhanced expression of interleukin (IL)-1 and IL-6 messenger RNA and bioactive protein after hypoxia-ischemia in neonatal rats. *Pediatr. Res.* 40 603–609. 10.1203/00006450-199610000-00015 8888290

[B111] Hagberg H. Mallard C. Ferriero D. M. Vannucci S. J. Levison S. W. Vexler Z. S. (2015). The role of inflammation in perinatal brain injury. *Nat. Rev. Neurol.* 11 192–208. 25686754 10.1038/nrneurol.2015.13 PMC4664161

[B112] Hajj G. N. Arantes C. P. Dias M. V. Roffé M. Costa-Silva B. Lopes M. H. (2013). The unconventional secretion of stress-inducible protein 1 by a heterogeneous population of extracellular vesicles. *Cell. Mol. Life Sci.* 70 3211–3227. 10.1007/s00018-013-1328-y 23543276 PMC11113396

[B113] Hammad A. Westacott L. Zaben M. (2018). The role of the complement system in traumatic brain injury: a review. *J. Neuroinflammation* 15:24. 10.1186/s12974-018-1066-z PMC5778697 29357880

[B114] Han J. Yang S. Hao X. Zhang B. Zhang H. Xin C. (2020). Extracellular vesicle-derived microRNA-410 from mesenchymal stem cells protects against neonatal hypoxia-ischemia brain damage through an HDAC1-dependent EGR2/Bcl2 Axis. *Front. Cell Dev. Biol.* 8:579236. 10.3389/fcell.2020.579236 33505958 PMC7829500

[B115] Hanson L. R. Frey W. H. II (2008). Intranasal delivery bypasses the blood-brain barrier to target therapeutic agents to the central nervous system and treat neurodegenerative disease. *BMC Neurosci.* 9(Suppl. 3):S5. 10.1186/1471-2202-9-S3-S5 19091002 PMC2604883

[B116] Huang Y. Liu Z. Tan F. Hu Z. Lu M. (2020). Effects of the insulted neuronal cells-derived extracellular vesicles on the survival of umbilical cord-derived mesenchymal stem cells following cerebral ischemia/reperfusion injury. *Oxid. Med. Cell. Longev.* 2020:9768713. 10.1155/2020/9768713 PMC7382764 32724498

[B117] Hunter M. P. Ismail N. Zhang X. Aguda B. D. Lee E. J. Yu L. (2008). Detection of microRNA expression in human peripheral blood microvesicles. *PLoS One* 3:e3694. 10.1371/journal.pone.0003694 19002258 PMC2577891

[B118] Iadecola C. Anrather J. (2011). The immunology of stroke: from mechanisms to translation. *Nat. Med.* 17 796–808. 10.1038/nm.2399 21738161 PMC3137275

[B119] Ikeda T. (2008). Stem cells and neonatal brain injury. *Cell Tissue Res.* 331 263–269. 10.1007/s00441-007-0546-8 18040721

[B120] Ilancheran S. Michalska A. Peh G. Wallace E. M. Pera M. Manuelpillai U. (2007). Stem cells derived from human fetal membranes display multi-lineage differentiation potential. *Biol. Reprod.* 77 577–588. 10.1095/biolreprod.106.055244 17494917

[B121] Inder T. E. Anderson N. J. Spencer C. Wells S. Volpe J. J. (2003a). White matter injury in the premature infant: a comparison between serial cranial sonographic and MR findings at term. *AJNR Am. J. Neuroradiol.* 24 805–809. 12748075 PMC7975772

[B122] Inder T. E. Buckland L. Williams C. E. Spencer C. Gunning M. I. Darlow B. A. (2003b). Lowered electroencephalographic spectral edge frequency predicts the presence of cerebral white matter injury in premature infants. *Pediatrics* 111 27–33. 10.1542/peds.111.1.27 12509550

[B123] Inder T. E. Huppi P. S. Warfield S. Kikinis R. Zientara G. P. Barnes P. D. (1999). Periventricular white matter injury in the premature infant is followed by reduced cerebral cortical gray matter volume at term. *Ann. Neurol.* 46 755–760. 10.1002/1531-8249(199911)46:5<755::aid-ana11>3.0.co;2-0 10553993

[B124] Inder T. E. Warfield S. K. Wang H. Hüppi P. S. Volpe J. J. (2005). Abnormal cerebral structure is present at term in premature infants. *Pediatrics* 115 286–294. 10.1542/peds.2004-0326 15687434

[B125] Irmady K. Jackman K. A. Padow V. A. Shahani N. Martin L. A. Cerchietti L. (2014). Mir-592 regulates the induction and cell death-promoting activity of p75NTR in neuronal ischemic injury. *J. Neurosci.* 34 3419–3428. 10.1523/jneurosci.1982-13.2014 24573298 PMC3935094

[B126] Izadpanah R. Kaushal D. Kriedt C. Tsien F. Patel B. Dufour J. (2008). Long-term in vitro expansion alters the biology of adult mesenchymal stem cells. *Cancer Res.* 68 4229–4238. 10.1158/0008-5472.can-07-5272 18519682 PMC2713721

[B127] Jacobs S. E. Berg M. Hunt R. Tarnow-Mordi W. O. Inder T. E. Davis P. G. (2013). Cooling for newborns with hypoxic ischaemic encephalopathy. *Cochrane Database Syst. Rev.* 2013:CD003311. 10.1002/14651858.CD003311 14583966

[B128] Jacobs S. E. Morley C. J. Inder T. E. Stewart M. J. Smith K. R. Mcnamara P. J. (2011). Whole-body hypothermia for term and near-term newborns with hypoxic-ischemic encephalopathy: a randomized controlled trial. *Arch. Pediatr. Adolesc. Med.* 165 692–700. 10.1001/archpediatrics.2011.43 21464374

[B129] Jarmalavičiūtė A. Tunaitis V. Pivoraitė U. Venalis A. Pivoriūnas A. (2015). Exosomes from dental pulp stem cells rescue human dopaminergic neurons from 6-hydroxy-dopamine-induced apoptosis. *Cytotherapy* 17 932–939. 10.1016/j.jcyt.2014.07.013 25981557

[B130] Jellema R. K. Lima Passos V. Zwanenburg A. Ophelders D. R. De Munter S. Vanderlocht J. (2013a). Cerebral inflammation and mobilization of the peripheral immune system following global hypoxia-ischemia in preterm sheep. *J. Neuroinflammation* 10:13. 10.1111/jpc.13882_26 PMC3614445 23347579

[B131] Jellema R. K. Wolfs T. G. Lima Passos V. Zwanenburg A. Ophelders D. R. Kuypers E. (2013b). Mesenchymal stem cells induce T-cell tolerance and protect the preterm brain after global hypoxia-ischemia. *PLoS One* 8:e73031. 10.1371/journal.pone.0073031 23991170 PMC3753351

[B132] Jeppesen D. K. Fenix A. M. Franklin J. L. Higginbotham J. N. Zhang Q. Zimmerman L. J. (2019). Reassessment of exosome composition. *Cell* 177 428–445.e418. 30951670 10.1016/j.cell.2019.02.029 PMC6664447

[B133] Jiang Y. Zhu J. Xu G. Liu X. (2011). Intranasal delivery of stem cells to the brain. *Expert Opin. Drug Deliv.* 8 623–632. 10.1517/17425247.2011.566267 21417782

[B134] Joerger-Messerli M. S. Oppliger B. Spinelli M. Thomi G. Di Salvo I. Schneider P. (2018). Extracellular vesicles derived from Wharton’s jelly mesenchymal stem cells prevent and resolve programmed cell death mediated by perinatal hypoxia-ischemia in neuronal cells. *Cell Transplant.* 27 168–180. 10.1177/0963689717738256 29562785 PMC6434490

[B135] Joshi P. Turola E. Ruiz A. Bergami A. Libera D. D. Benussi L. (2014). Microglia convert aggregated amyloid-β into neurotoxic forms through the shedding of microvesicles. *Cell Death Differ.* 21 582–593. 10.1038/cdd.2013.180 24336048 PMC3950321

[B136] Juul S. E. Pet G. C. (2015). Erythropoietin and neonatal neuroprotection. *Clin. Perinatol.* 42 469–481. 10.1016/j.clp.2015.04.004 26250911 PMC4529536

[B137] Kadhim H. Tabarki B. Verellen G. De Prez C. Rona A. M. Sébire G. (2001). Inflammatory cytokines in the pathogenesis of periventricular leukomalacia. *Neurology* 56 1278–1284. 10.1212/wnl.56.10.1278 11376173

[B138] Kalra H. Simpson R. J. Ji H. Aikawa E. Altevogt P. Askenase P. (2012). Vesiclepedia: a compendium for extracellular vesicles with continuous community annotation. *PLoS Biol.* 10:e1001450. 10.1371/journal.pbio.1001450 23271954 PMC3525526

[B139] Kaminski N. Köster C. Mouloud Y. Börger V. Felderhoff-Müser U. Bendix I. (2020). Mesenchymal stromal cell-derived extracellular vesicles reduce neuroinflammation, promote neural cell proliferation and improve oligodendrocyte maturation in neonatal hypoxic-ischemic brain injury. *Front. Cell. Neurosci.* 14:601176. 10.3389/fncel.2020.601176 33362471 PMC7758466

[B140] Kennea N. L. Mehmet H. (2004). Perinatal applications of neural stem cells. *Best Pract. Res. Clin. Obstet. Gynaecol.* 18 977–994. 15582550 10.1016/j.bpobgyn.2004.06.008

[B141] Keunen K. Kersbergen K. J. Groenendaal F. Isgum I. De Vries L. S. Benders M. J. (2012). Brain tissue volumes in preterm infants: prematurity, perinatal risk factors and neurodevelopmental outcome: a systematic review. *J. Matern. Fetal Neonatal Med.* 25 89–100. 10.3109/14767058.2012.664343 22348253

[B142] Khwaja O. Volpe J. J. (2008). Pathogenesis of cerebral white matter injury of prematurity. *Arch. Dis. Child. Fetal Neonatal Ed.* 93 F153–F161. 18296574 10.1136/adc.2006.108837 PMC2569152

[B143] Kim E. S. Ahn S. Y. Im G. H. Sung D. K. Park Y. R. Choi S. H. (2012). Human umbilical cord blood–derived mesenchymal stem cell transplantation attenuates severe brain injury by permanent middle cerebral artery occlusion in newborn rats. *Pediatr. Res.* 72 277–284. 10.1038/pr.2012.71 22669296

[B144] Kinney H. C. Haynes R. L. Xu G. Andiman S. E. Folkerth R. D. Sleeper L. A. (2012). Neuron deficit in the white matter and subplate in periventricular leukomalacia. *Ann. Neurol.* 71 397–406. 10.1002/ana.22612 22451205 PMC3315053

[B145] Ko H. R. Ahn S. Y. Chang Y. S. Hwang I. Yun T. Sung D. K. (2018). Human UCB-MSCs treatment upon intraventricular hemorrhage contributes to attenuate hippocampal neuron loss and circuit damage through BDNF-CREB signaling. *Stem Cell Res. Ther.* 9:326. 10.1186/s13287-018-1052-5 PMC6249960 30463591

[B146] Kodali M. Castro O. W. Kim D.-K. Thomas A. Shuai B. Attaluri S. (2019). Intranasally administered human MSC-derived extracellular vesicles pervasively incorporate into neurons and microglia in both intact and status epilepticus injured forebrain. *Int. J. Mol. Sci.* 21:181. 10.3390/ijms21010181 31888012 PMC6981466

[B147] Kooijmans S. A. A. Fliervoet L. A. L. Van Der Meel R. Fens M. Heijnen H. F. G. Van Bergen En Henegouwen P. M. P. (2016). PEGylated and targeted extracellular vesicles display enhanced cell specificity and circulation time. *J. Control. Release* 224 77–85. 10.1016/j.jconrel.2016.01.009 26773767

[B148] Korkut C. Li Y. Koles K. Brewer C. Ashley J. Yoshihara M. (2013). Regulation of postsynaptic retrograde signaling by presynaptic exosome release. *Neuron* 77 1039–1046. 10.1016/j.neuron.2013.01.013 23522040 PMC3626103

[B149] Krämer-Albers E. M. Bretz N. Tenzer S. Winterstein C. Möbius W. Berger H. (2007). Oligodendrocytes secrete exosomes containing major myelin and stress-protective proteins: trophic support for axons? *Proteomics Clin. Appl.* 1 1446–1461. 10.1002/prca.200700522 21136642

[B150] Kumar A. Mittal R. Khanna H. D. Basu S. (2008). Free radical injury and blood-brain barrier permeability in hypoxic-ischemic encephalopathy. *Pediatrics* 122 e722–e727. 18725389 10.1542/peds.2008-0269

[B151] Lappalainen R. S. Narkilahti S. Huhtala T. Liimatainen T. Suuronen T. Narvanen A. (2008). The SPECT imaging shows the accumulation of neural progenitor cells into internal organs after systemic administration in middle cerebral artery occlusion rats. *Neurosci Lett* 440 246–250. 10.1016/j.neulet.2008.05.090 18572314

[B152] Lau L. T. Yu A. C. (2001). Astrocytes produce and release interleukin-1, interleukin-6, tumor necrosis factor alpha and interferon-gamma following traumatic and metabolic injury. *J. Neurotrauma* 18 351–359. 10.1089/08977150151071035 11284554

[B153] Lee J. Y. Kim H.-S. (2017). Extracellular vesicles in neurodegenerative diseases: a double-edged sword. *Tissue Eng. Regen. Med.* 14 667–678. 10.1007/s13770-017-0090-x 30603519 PMC6171665

[B154] Lee S. J. Hatran D. P. Tomimatsu T. Peña J. P. Mcauley G. Longo L. D. (2009). Fetal cerebral blood flow, electrocorticographic activity, and oxygenation: responses to acute hypoxia. *J. Physiol.* 587 2033–2047. 10.1113/jphysiol.2009.166983 19406885 PMC2689341

[B155] Lee W. L. A. Michael-Titus A. T. Shah D. K. (2017). Hypoxic-Ischaemic encephalopathy and the blood-brain barrier in neonates. *Dev. Neurosci.* 39 49–58. 10.1159/000467392 28434009

[B156] Lener T. Gimona M. Aigner L. Börger V. Buzas E. Camussi G. (2015). Applying extracellular vesicles based therapeutics in clinical trials - an ISEV position paper. *J. Extracell. Vesicles* 4:30087. 10.3402/jev.v4.30087 PMC4698466 26725829

[B157] Leuchter R. H. Gui L. Poncet A. Hagmann C. Lodygensky G. A. Martin E. (2014). Association between early administration of high-dose erythropoietin in preterm infants and brain MRI abnormality at term-equivalent age. *JAMA* 312 817–824. 10.1001/jama.2014.9645 25157725

[B158] Leviton A. Gressens P. (2007). Neuronal damage accompanies perinatal white-matter damage. *Trends Neurosci.* 30 473–478. 10.1016/j.tins.2007.05.009 17765331

[B159] Lewis S. (2013). Glia: transporting cargo from A to B. *Nat. Rev. Neurosci.* 14:589. 10.1038/nrn3568 23900413

[B160] Li H. Niederkorn J. Neelam S. Mayhew E. Word R. Mcculley J. (2005). Immunosuppressive factors secreted by human amniotic epithelial cells. *Invest. Ophthalmol. Vis. Sci.* 46 900–907. 10.1167/iovs.04-0495 15728546

[B161] Lieberman M. A. Glaser L. (1981). Density-dependent regulation of cell growth: an example of a cell-cell recognition phenomenon. *J. Membr. Biol.* 63 1–11. 10.1007/bf01969440 6273565

[B162] Liu W. Wang Y. Gong F. Rong Y. Luo Y. Tang P. (2019). Exosomes derived from bone mesenchymal stem cells repair traumatic spinal cord injury by suppressing the activation of A1 neurotoxic reactive astrocytes. *J. Neurotrauma* 36 469–484. 10.1089/neu.2018.5835 29848167

[B163] Lombardi M. Parolisi R. Scaroni F. Bonfanti E. Gualerzi A. Gabrielli M. (2019). Detrimental and protective action of microglial extracellular vesicles on myelin lesions: astrocyte involvement in remyelination failure. *Acta Neuropathol.* 138 987–1012. 10.1007/s00401-019-02049-1 31363836 PMC6851224

[B164] Luarte A. Batiz L. F. Wyneken U. Lafourcade C. (2016). Potential therapies by stem cell-derived exosomes in CNS diseases: focusing on the neurogenic niche. *Stem Cells Int.* 2016:5736059. 10.1155/2016/5736059 PMC4853949 27195011

[B165] Lutz I. C. Allegaert K. De Hoon J. N. Marynissen H. (2020). Pharmacokinetics during therapeutic hypothermia for neonatal hypoxic ischaemic encephalopathy: a literature review. *BMJ Paediatr. Open* 4:e000685. 10.1136/bmjpo-2020-000685 32577535 PMC7299043

[B166] Ma S. Xie N. Li W. Yuan B. Shi Y. Wang Y. (2014). Immunobiology of mesenchymal stem cells. *Cell Death Differ.* 21 216–225. 24185619 10.1038/cdd.2013.158 PMC3890955

[B167] Ma Y. Wang K. Pan J. Fan Z. Tian C. Deng X. (2019). Induced neural progenitor cells abundantly secrete extracellular vesicles and promote the proliferation of neural progenitors via extracellular signal-regulated kinase pathways. *Neurobiol. Dis.* 124 322–334. 10.1016/j.nbd.2018.12.003 30528256 PMC6450400

[B168] Mallard C. Davidson J. O. Tan S. Green C. R. Bennet L. Robertson N. J. (2014). Astrocytes and microglia in acute cerebral injury underlying cerebral palsy associated with preterm birth. *Pediatr. Res.* 75 234–240. 10.1038/pr.2013.188 24336433 PMC11908707

[B169] Manaenko A. Lekic T. Barnhart M. Hartman R. Zhang J. H. (2014). Inhibition of transforming growth factor-β attenuates brain injury and neurological deficits in a rat model of germinal matrix hemorrhage. *Stroke* 45 828–834. 10.1161/strokeaha.113.003754 24425124 PMC3966308

[B170] March of Dimes, Pmnch, Save the Children Who. (2012). in *Born Too Soon: The Global Action Report on Preterm Birth*, eds Howson C. Kinney M. Lawn J. Geneva: World Health Organisation.

[B171] Marcus M. E. Leonard J. N. (2013). FedExosomes: engineering therapeutic biological nanoparticles that truly deliver. *Pharmaceuticals (Basel, Switzerland)* 6 659–680. 10.3390/ph6050659 23894228 PMC3722064

[B172] Margolis L. Sadovsky Y. (2019). The biology of extracellular vesicles: the known unknowns. *PLoS Biol.* 17:e3000363. 10.1371/journal.pbio.3000363 31318874 PMC6667152

[B173] Martin C. R. Dammann O. Allred E. N. Patel S. O’shea T. M. Kuban K. C. (2010). Neurodevelopment of extremely preterm infants who had necrotizing enterocolitis with or without late bacteremia. *J. Pediatr.* 157 751–756.e751. 20598317 10.1016/j.jpeds.2010.05.042 PMC2952050

[B174] Mateescu B. Kowal E. J. Van Balkom B. W. Bartel S. Bhattacharyya S. N. Buzás E. I. (2017). Obstacles and opportunities in the functional analysis of extracellular vesicle RNA - an ISEV position paper. *J. Extracell. Vesicles* 6:1286095. 10.1080/20013078.2017.1286095 28326170 PMC5345583

[B175] Mathieu M. Martin-Jaular L. Lavieu G. Thery C. (2019). Specificities of secretion and uptake of exosomes and other extracellular vesicles for cell-to-cell communication. *Nat. Cell Biol.* 21 9–17. 10.1038/s41556-018-0250-9 30602770

[B176] Matsumoto J. Stewart T. Sheng L. Li N. Bullock K. Song N. (2017). Transmission of alpha-synuclein-containing erythrocyte-derived extracellular vesicles across the blood-brain barrier via adsorptive mediated transcytosis: another mechanism for initiation and progression of Parkinson’s disease? *Acta Neuropathol. Commun.* 5:71. 10.1186/s40478-017-0470-4 PMC5598000 28903781

[B177] McNamara N. B. Miron V. E. (2020). Microglia in developing white matter and perinatal brain injury. *Neurosci. Lett.* 714:134539. 10.1016/j.neulet.2019.134539 31614181

[B178] Merkus F. W. H. M. van den Berg M. P. (2007). Can nasal drug delivery bypass the blood-brain barrier? *Drugs R D* 8 133–144. 10.2165/00126839-200708030-00001 17472409

[B179] Miller S. P. Cozzio C. C. Goldstein R. B. Ferriero D. M. Partridge J. C. Vigneron D. B. (2003). Comparing the diagnosis of white matter injury in premature newborns with serial MR imaging and transfontanel ultrasonography findings. *AJNR Am. J. Neuroradiol.* 24 1661–1669. 13679289 PMC7973994

[B180] Mo M. Wang S. Zhou Y. Li H. Wu Y. (2016). Mesenchymal stem cell subpopulations: phenotype, property and therapeutic potential. *Cell. Mol. Life Sci.* 73 3311–3321. 10.1007/s00018-016-2229-7 27141940 PMC11108490

[B181] Mondello S. Thelin E. P. Shaw G. Salzet M. Visalli C. Cizkova D. (2018). Extracellular vesicles: pathogenetic, diagnostic and therapeutic value in traumatic brain injury. *Expert Rev. Proteomics* 15 451–461. 10.1080/14789450.2018.1464914 29671356

[B182] Morton M. C. Feliciano D. M. (2016). Neurovesicles in brain development. *Cell. Mol. Neurobiol.* 36 409–416. 10.1007/s10571-015-0297-0 26993505 PMC11482443

[B183] Morton M. C. Neckles V. N. Seluzicki C. M. Holmberg J. C. Feliciano D. M. (2018). Neonatal subventricular zone neural stem cells release extracellular vesicles that act as a microglial morphogen. *Cell Rep.* 23 78–89. 10.1016/j.celrep.2018.03.037 29617675

[B184] Murphy D. E. De Jong O. G. Brouwer M. Wood M. J. Lavieu G. Schiffelers R. M. (2019). Extracellular vesicle-based therapeutics: natural versus engineered targeting and trafficking. *Exp. Mol. Med.* 51 1–12. 10.1038/s12276-019-0223-5 PMC6418170 30872574

[B185] Murphy S. Lim R. Dickinson H. Acharya R. Rosli S. Jenkin G. (2010). Human amnion epithelial cells prevent bleomycin-induced lung injury and preserve lung function. *Cell Transplant.* 19 909–923. 10.3727/096368910x543385 21092408

[B186] Murray A. L. Thompson D. K. Pascoe L. Leemans A. Inder T. E. Doyle L. W. (2016). White matter abnormalities and impaired attention abilities in children born very preterm. *Neuroimage* 124 75–84. 10.1016/j.neuroimage.2015.08.044 26318524 PMC4791057

[B187] Murray D. M. Boylan G. B. Ryan C. A. Connolly S. (2009). Early EEG findings in hypoxic-ischemic encephalopathy predict outcomes at 2 years. *Pediatrics* 124 e459–e467. 19706569 10.1542/peds.2008-2190

[B188] Naeye R. L. Lin H. M. (2001). Determination of the timing of fetal brain damage from hypoxemia-ischemia. *Am. J. Obstet. Gynecol.* 184 217–224. 10.1067/mob.2001.108996 11174505

[B189] Nakamura K. Arimura K. Nishimura A. Tachibana M. Yoshikawa Y. Makihara N. (2016). Possible involvement of basic FGF in the upregulation of PDGFRβ in pericytes after ischemic stroke. *Brain Res.* 1630 98–108. 10.1016/j.brainres.2015.11.003 26569132

[B190] Nishida-Aoki N. Izumi Y. Takeda H. Takahashi M. Ochiya T. Bamba T. (2020). Lipidomic analysis of cells and extracellular vesicles from high- and low-metastatic triple-negative breast cancer. *Metabolites* 10:67. 10.3390/metabo10020067 32069969 PMC7073695

[B191] Ohmichi T. Mitsuhashi M. Tatebe H. Kasai T. Ali El-Agnaf O. M. Tokuda T. (2019). Quantification of brain-derived extracellular vesicles in plasma as a biomarker to diagnose Parkinson’s and related diseases. *Parkinsonism Relat. Disord.* 61 82–87. 10.1016/j.parkreldis.2018.11.021 30502924

[B192] Ophelders D. R. M. G. Gussenhoven R. Klein L. Jellema R. K. Westerlaken R. J. J. Hütten M. C. (2020). Preterm brain injury, antenatal triggers, and therapeutics: timing is key. *Cells* 9:1871. 10.3390/cells9081871 32785181 PMC7464163

[B193] Ophelders D. R. Wolfs T. G. Jellema R. K. Zwanenburg A. Andriessen P. Delhaas T. (2016). Mesenchymal stromal cell-derived extracellular vesicles protect the fetal brain after hypoxia-ischemia. *Stem Cells Transl. Med.* 5 754–763. 10.5966/sctm.2015-0197 27160705 PMC4878333

[B194] Oskoui M. Coutinho F. Dykeman J. Jetté N. Pringsheim T. (2013). An update on the prevalence of cerebral palsy: a systematic review and meta-analysis. *Dev. Med. Child Neurol.* 55 509–519. 10.1111/dmcn.12080 23346889

[B195] Pardridge W. M. (2012). Drug transport across the blood-brain barrier. *J. Cereb. Blood Flow Metab.* 32 1959–1972. 22929442 10.1038/jcbfm.2012.126 PMC3494002

[B196] Park H. Park H. Mun D. Kang J. Kim H. Kim M. (2018). Extracellular vesicles derived from hypoxic human mesenchymal stem cells attenuate GSK3β expression via miRNA-26a in an ischemia-reperfusion injury model. *Yonsei Med. J.* 59 736–745. 10.3349/ymj.2018.59.6.736 29978610 PMC6037597

[B197] Patel D. B. Gray K. M. Santharam Y. Lamichhane T. N. Stroka K. M. Jay S. M. (2017). Impact of cell culture parameters on production and vascularization bioactivity of mesenchymal stem cell-derived extracellular vesicles. *Bioeng. Transl. Med.* 2 170–179. 10.1002/btm2.10065 28932818 PMC5579732

[B198] Patel D. B. Santoro M. Born L. J. Fisher J. P. Jay S. M. (2018). Towards rationally designed biomanufacturing of therapeutic extracellular vesicles: impact of the bioproduction microenvironment. *Biotechnol. Adv.* 36 2051–2059. 10.1016/j.biotechadv.2018.09.001 30218694 PMC6250573

[B199] Penn A. A. Gressens P. Fleiss B. Back S. A. Gallo V. (2016). Controversies in preterm brain injury. *Neurobiol. Dis.* 92 90–101. 10.1016/j.nbd.2015.10.012 26477300 PMC4842157

[B200] Pierson C. R. Folkerth R. D. Billiards S. S. Trachtenberg F. L. Drinkwater M. E. Volpe J. J. (2007). Gray matter injury associated with periventricular leukomalacia in the premature infant. *Acta Neuropathol.* 114 619–631. 10.1007/s00401-007-0295-5 17912538 PMC2080348

[B201] Pimentel-Coelho P. M. Mendez-Otero R. (2010). Cell therapy for neonatal hypoxic-ischemic encephalopathy. *Stem Cells Dev.* 19 299–310. 19916801 10.1089/scd.2009.0403

[B202] Pimentel-Coelho P. M. Magalhaes E. S. Lopes L. M. Deazevedo L. C. Santiago M. F. Mendez-Otero R. (2010). Human cord blood transplantation in a neonatal rat model of hypoxic-ischemic brain damage: functional outcome related to neuroprotection in the striatum. *Stem Cells Dev.* 19 351–358. 10.1089/scd.2009.0049 19296724

[B203] Platt M. J. Cans C. Johnson A. Surman G. Topp M. Torrioli M. G. (2007). Trends in cerebral palsy among infants of very low birthweight (<1500 g) or born prematurely (<32 weeks) in 16 European centres: a database study. *Lancet* 369 43–50. 10.1016/s0140-6736(07)60030-0 17208641

[B204] Polanco J. C. Scicluna B. J. Hill A. F. Götz J. (2016). Extracellular vesicles isolated from the brains of rTg4510 mice seed tau protein aggregation in a threshold-dependent manner. *J. Biol. Chem.* 291 12445–12466. 10.1074/jbc.m115.709485 27030011 PMC4933440

[B205] Polglase G. R. Miller S. L. Barton S. K. Baburamani A. A. Wong F. Y. Aridas J. D. (2012). Initiation of resuscitation with high tidal volumes causes cerebral hemodynamic disturbance, brain inflammation and injury in preterm lambs. *PLoS One* 7:e39535. 10.1371/journal.pone.0039535 22761816 PMC3382197

[B206] Pollet H. Conrard L. Cloos A. S. Tyteca D. (2018). Plasma membrane lipid domains as platforms for vesicle biogenesis and shedding? *Biomolecules* 8:94. 10.3390/biom8030094 30223513 PMC6164003

[B207] Prada I. Gabrielli M. Turola E. Iorio A. D’arrigo G. Parolisi R. (2018). Glia-to-neuron transfer of miRNAs via extracellular vesicles: a new mechanism underlying inflammation-induced synaptic alterations. *Acta Neuropathol.* 135 529–550. 10.1007/s00401-017-1803-x 29302779 PMC5978931

[B208] Rajendran L. Honsho M. Zahn T. R. Keller P. Geiger K. D. Verkade P. (2006). Alzheimer’s disease β-amyloid peptides are released in association with exosomes. *Proc. Natl. Acad. Sci. U.S.A.* 103 11172–11177. 10.1073/pnas.0603838103 16837572 PMC1544060

[B209] Ranasinghe H. S. Scheepens A. Sirimanne E. Mitchell M. D. Williams C. E. Fraser M. (2012). Inhibition of MMP-9 activity following hypoxic ischemia in the developing brain using a highly specific inhibitor. *Dev. Neurosci.* 34 417–427. 10.1159/000343257 23171520

[B210] Ranasinghe H. S. Williams C. E. Christophidis L. J. Mitchell M. D. Fraser M. Scheepens A. (2009). Proteolytic activity during cortical development is distinct from that involved in hypoxic ischemic injury. *Neuroscience* 158 732–744. 18809469 10.1016/j.neuroscience.2008.07.069

[B211] Ratajczak M. Z. Kucia M. Jadczyk T. Greco N. J. Wojakowski W. Tendera M. (2012). Pivotal role of paracrine effects in stem cell therapies in regenerative medicine: can we translate stem cell-secreted paracrine factors and microvesicles into better therapeutic strategies? *Leukemia* 26 1166–1173. 10.1038/leu.2011.389 22182853

[B212] Reddy K. Mallard C. Guan J. Marks K. Bennet L. Gunning M. (1998). Maturational Change in the Cortical Response to Hypoperfusion Injury in the Fetal Sheep. *Pediatr. Res.* 43 674–682. 10.1203/00006450-199805000-00017 9585015

[B213] Rezaie P. Dean A. (2002). Periventricular leukomalacia, inflammation and white matter lesions within the developing nervous system. *Neuropathology* 22 106–132. 10.1046/j.1440-1789.2002.00438.x 12416551

[B214] Robertson C. M. Watt M. J. Yasui Y. (2007). Changes in the prevalence of cerebral palsy for children born very prematurely within a population-based program over 30 years. *JAMA* 297 2733–2740. 10.1001/jama.297.24.2733 17595274

[B215] Robey P. (2017). “Mesenchymal stem cells”: fact or fiction, and implications in their therapeutic use. *F1000Res* 6:524. 10.12688/f1000research.10955.1 28491279 PMC5399967

[B216] Rutherford M. A. Supramaniam V. Ederies A. Chew A. Bassi L. Groppo M. (2010). Magnetic resonance imaging of white matter diseases of prematurity. *Neuroradiology* 52 505–521. 20422407 10.1007/s00234-010-0700-y

[B217] Saint-Pol J. Gosselet F. Duban-Deweer S. Pottiez G. Karamanos Y. (2020). Targeting and crossing the blood-brain barrier with extracellular vesicles. *Cells* 9:851. 10.3390/cells9040851 32244730 PMC7226770

[B218] Santner-Nanan B. Peek M. J. Mccullagh P. Nanan R. (2005). Therapeutic potential of stem cells in perinatal medicine. *Aust. N. Z. J. Obstet. Gynaecol.* 45 102–107. 15760308 10.1111/j.1479-828X.2005.00362.x

[B219] Schmahmann J. Pandya D. (2006). *Fibre Pathways of the Brain.* New York, NY: Oxford University Press.

[B220] Schneider J. Miller S. P. (2019). Preterm brain Injury: white matter injury. *Handb. Clin. Neurol.* 162 155–172. 10.1016/b978-0-444-64029-1.00007-2 31324309

[B221] Seifert H. A. Leonardo C. C. Hall A. A. Rowe D. D. Collier L. A. Benkovic S. A. (2012). The spleen contributes to stroke induced neurodegeneration through interferon gamma signaling. *Metab. Brain Dis.* 27 131–141. 10.1007/s11011-012-9283-0 22354752 PMC4739736

[B222] Shankaran S. Laptook A. R. Tyson J. E. Ehrenkranz R. A. Bann C. M. Das A. (2012). Evolution of encephalopathy during whole body hypothermia for neonatal hypoxic-ischemic encephalopathy. *J. Pediatr.* 160 567–572.e563. 22050871 10.1016/j.jpeds.2011.09.018 PMC3299861

[B223] Sharma P. Mesci P. Carromeu C. Mcclatchy D. R. Schiapparelli L. Yates J. R. (2019). Exosomes regulate neurogenesis and circuit assembly. *Proc. Natl. Acad. Sci. U.S.A.* 116:16086. 10.1073/pnas.1902513116 31320591 PMC6689941

[B224] Sharma P. Schiapparelli L. Cline H. T. (2013). Exosomes function in cell-cell communication during brain circuit development. *Curr. Opin. Neurobiol.* 23 997–1004. 10.1016/j.conb.2013.08.005 23998929 PMC3830597

[B225] Sheller-Miller S. Lei J. Saade G. Salomon C. Burd I. Menon R. (2016). Feto-Maternal trafficking of exosomes in murine pregnancy models. *Front. Pharmacol.* 7:432. 10.3389/fphar.2016.00432 27895585 PMC5108780

[B226] Shu J. Mo S. Wen Q. Qin Y. Jiang L. (2018). Study on immune regulation of bone marrow mesenchymal stem cell-derived exosomes in preterm infants with brain injury. *Nanosci. Nanotechnol. Lett.* 10 1598–1605. 10.1166/nnl.2018.2824

[B227] Simpson R. J. Jensen S. S. Lim J. W. (2008). Proteomic profiling of exosomes: current perspectives. *Proteomics* 8 4083–4099. 10.1002/pmic.200800109 18780348

[B228] Sisa C. Kholia S. Naylor J. Herrera Sanchez M. B. Bruno S. Deregibus M. C. (2019). Mesenchymal stromal cell derived extracellular vesicles reduce hypoxia-ischaemia induced perinatal brain injury. *Front. Physiol.* 10:282. 10.3389/fphys.2019.00282 30941062 PMC6433879

[B229] Skiöld B. Wu Q. Hooper S. B. Davis P. G. Mcintyre R. Tolcos M. (2014). Early detection of ventilation-induced brain injury using magnetic resonance spectroscopy and diffusion tensor imaging: an in vivo study in preterm lambs. *PLoS One* 9:e95804. 10.1371/journal.pone.0095804 24759765 PMC3997476

[B230] Skotland T. Sagini K. Sandvig K. Llorente A. (2020). An emerging focus on lipids in extracellular vesicles. *Adv. Drug Deliv. Rev.* 159 308–321. 10.1016/j.addr.2020.03.002 32151658

[B231] Skranes J. Løhaugen G. C. Martinussen M. Håberg A. Brubakk A. M. Dale A. M. (2013). Cortical surface area and IQ in very-low-birth-weight (VLBW) young adults. *Cortex* 49 2264–2271. 10.1016/j.cortex.2013.06.001 23845237

[B232] Smith Z. J. Lee C. Rojalin T. Carney R. P. Hazari S. Knudson A. (2015). Single exosome study reveals subpopulations distributed among cell lines with variability related to membrane content. *J. Extracell. Vesicles* 4:28533. 10.3402/jev.v4.28533 26649679 PMC4673914

[B233] Somiya M. Yoshioka Y. Ochiya T. (2017). Drug delivery application of extracellular vesicles; insight into production, drug loading, targeting, and pharmacokinetics. *AIMS Bioeng.* 4 73–92.

[B234] Spaull R. Mcpherson B. Gialeli A. Clayton A. Uney J. Heep A. (2019). Exosomes populate the cerebrospinal fluid of preterm infants with post-haemorrhagic hydrocephalus. *Int. J. Dev. Neurosci.* 73 59–65. 10.1016/j.ijdevneu.2019.01.004 30639393

[B235] Stoll B. J. Hansen N. I. Adams-Chapman I. Fanaroff A. A. Hintz S. R. Vohr B. (2004). Neurodevelopmental and growth impairment among extremely low-birth-weight infants with neonatal infection. *JAMA* 292 2357–2365. 10.1001/jama.292.19.2357 15547163

[B236] Sun L. Q. Guo G. L. Zhang S. Yang L. L. (2018). Effects of MicroRNA-592-5p on hippocampal neuron injury following hypoxic-ischemic brain damage in neonatal mice - involvement of PGD2/DP and PTGDR. *Cell. Physiol. Biochem.* 45 458–473. 10.1159/000486923 29402808

[B237] Takenouchi T. Tsukimoto M. Iwamaru Y. Sugama S. Sekiyama K. Sato M. (2015). Extracellular ATP induces unconventional release of glyceraldehyde-3-phosphate dehydrogenase from microglial cells. *Immunol. Lett.* 167 116–124. 10.1016/j.imlet.2015.08.002 26277554

[B238] Taylor D. D. Shah S. (2015). Methods of isolating extracellular vesicles impact down-stream analyses of their cargoes. *Methods* 87 3–10. 10.1016/j.ymeth.2015.02.019 25766927

[B239] Thery C. Witwer K. W. Aikawa E. Alcaraz M. J. Anderson J. D. Andriantsitohaina R. (2018). Minimal information for studies of extracellular vesicles 2018 (MISEV2018): a position statement of the International Society for Extracellular Vesicles and update of the MISEV2014 guidelines. *J. Extracell. Vesicles* 7:1535750. 10.1080/20013078.2018.1535750 PMC6322352 30637094

[B240] Théry C. Zitvogel L. Amigorena S. (2002). Exosomes: composition, biogenesis and function. *Nat. Rev. Immunol.* 2 569–579. 10.1038/nri855 12154376

[B241] Thomi G. Joerger-Messerli M. Haesler V. Muri L. Surbek D. Schoeberlein A. (2019a). Intranasally administered exosomes from umbilical cord stem cells have preventive neuroprotective effects and contribute to functional recovery after perinatal brain injury. *Cells* 8:855. 10.3390/cells8080855 31398924 PMC6721675

[B242] Thomi G. Surbek D. Haesler V. Joerger-Messerli M. Schoeberlein A. (2019b). Exosomes derived from umbilical cord mesenchymal stem cells reduce microglia-mediated neuroinflammation in perinatal brain injury. *Stem Cell Res. Ther.* 10:105. 10.1186/s13287-019-1207-z PMC6429800 30898154

[B243] Thompson A. G. Gray E. Heman-Ackah S. M. Mager I. Talbot K. Andaloussi S. E. (2016). Extracellular vesicles in neurodegenerative disease - pathogenesis to biomarkers. *Nat. Rev. Neurol.* 12 346–357. 10.1038/nrneurol.2016.68 27174238

[B244] Tietje A. Maron K. N. Wei Y. Feliciano D. M. (2014). Cerebrospinal fluid extracellular vesicles undergo age dependent declines and contain known and novel non-coding RNAs. *PLoS One* 9:e113116. 10.1371/journal.pone.0113116 25420022 PMC4242609

[B245] Valadi H. Ekström K. Bossios A. Sjöstrand M. Lee J. J. Lötvall J. O. (2007). Exosome-mediated transfer of mRNAs and microRNAs is a novel mechanism of genetic exchange between cells. *Nat. Cell Biol.* 9 654–659. 10.1038/ncb1596 17486113

[B246] van den Broek M. P. Groenendaal F. Egberts A. C. Rademaker C. M. (2010). Effects of hypothermia on pharmacokinetics and pharmacodynamics: a systematic review of preclinical and clinical studies. *Clin. Pharmacokinet.* 49 277–294. 10.2165/11319360-000000000-00000 20384391

[B247] van Haastert I. C. Groenendaal F. Uiterwaal C. S. Termote J. U. Van Der Heide-Jalving M. Eijsermans M. J. (2011). Decreasing incidence and severity of cerebral palsy in prematurely born children. *J. Pediatr.* 159 86–91.e81. 21367430 10.1016/j.jpeds.2010.12.053

[B248] van Tilborg E. De Theije C. G. M. Van Hal M. Wagenaar N. De Vries L. S. Benders M. J. (2018). Origin and dynamics of oligodendrocytes in the developing brain: implications for perinatal white matter injury. *Glia* 66 221–238. 10.1002/glia.23256 29134703 PMC5765410

[B249] van Tilborg E. Heijnen C. J. Benders M. J. Van Bel F. Fleiss B. Gressens P. (2016). Impaired oligodendrocyte maturation in preterm infants: potential therapeutic targets. *Prog. Neurobiol.* 136 28–49. 10.1016/j.pneurobio.2015.11.002 26655283

[B250] van Velthoven C. T. Kavelaars A. Heijnen C. J. (2012). Mesenchymal stem cells as a treatment for neonatal ischemic brain damage. *Pediatr. Res.* 71 474–481. 10.1038/pr.2011.64 22430383

[B251] van Velthoven C. T. Kavelaars A. Van Bel F. Heijnen C. J. (2010). Mesenchymal stem cell treatment after neonatal hypoxic-ischemic brain injury improves behavioral outcome and induces neuronal and oligodendrocyte regeneration. *Brain Behav. Immun.* 24 387–393. 10.1016/j.bbi.2009.10.017 19883750

[B252] Vannucci R. C. Vannucci S. J. (1997). A model of perinatal hypoxic-ischemic brain damage. *Ann. N. Y. Acad. Sci.* 835 234–249. 9616778 10.1111/j.1749-6632.1997.tb48634.x

[B253] Vawda R. Woodbury J. Covey M. Levison S. W. Mehmet H. (2007). Stem cell therapies for perinatal brain injuries. *Semin. Fetal Neonatal Med.* 12 259–272. 10.1016/j.siny.2007.02.003 17553762

[B254] Vincer M. J. Allen A. C. Joseph K. S. Stinson D. A. Scott H. Wood E. (2006). Increasing prevalence of cerebral palsy among very preterm infants: a population-based study. *Pediatrics* 118 e1621–e1626. 17074842 10.1542/peds.2006-1522

[B255] Vogel A. Upadhya R. Shetty A. K. (2018). Neural stem cell derived extracellular vesicles: attributes and prospects for treating neurodegenerative disorders. *EBioMedicine* 38 273–282. 10.1016/j.ebiom.2018.11.026 30472088 PMC6306394

[B256] Volpe J. J. Kinney H. C. Jensen F. E. Rosenberg P. A. (2011). The developing oligodendrocyte: key cellular target in brain injury in the premature infant. *Int. J. Dev. Neurosci.* 29 423–440. 10.1016/j.ijdevneu.2011.02.012 21382469 PMC3099053

[B257] Walsh W. (2015). Report of a pilot study of Cooling four preterm infants 32-35 weeks gestation with HIE. *J. Neonatal Perinatal Med.* 8 47–51. 10.3233/npm-15814078 25758006

[B258] Wang A. Y. Chughtai A. A. Lui K. Sullivan E. A. (2017). Morbidity and mortality among very preterm singletons following fertility treatment in Australia and New Zealand, a population cohort study. *BMC Pregnancy Childbirth* 17:50. 10.1186/s12884-017-1235-6 28148237 PMC5288897

[B259] Wang J.-X. Jia X.-J. Liu Y. Dong J.-H. Ren X.-M. Xu O. (2020). Silencing of miR-17-5p suppresses cell proliferation and promotes cell apoptosis by directly targeting PIK3R1 in laryngeal squamous cell carcinoma. *Cancer Cell Int.* 20:14. 10.1186/s12935-020-1096-3 PMC6954602 31938022

[B260] Wang X. Rousset C. I. Hagberg H. Mallard C. (2006). Lipopolysaccharide-induced inflammation and perinatal brain injury. *Semin. Fetal Neonatal Med.* 11 343–353. 10.1016/j.siny.2006.04.002 16793357

[B261] Wassink G. Davidson J. O. Dhillon S. K. Fraser M. Galinsky R. Bennet L. (2017). Partial white and grey matter protection with prolonged infusion of recombinant human erythropoietin after asphyxia in preterm fetal sheep. *J. Cereb. Blood Flow Metab.* 37 1080–1094. 10.1177/0271678x16650455 27207167 PMC5363482

[B262] Webb R. L. Kaiser E. E. Jurgielewicz B. J. Spellicy S. Scoville S. L. Thompson T. A. (2018a). Human neural stem cell extracellular vesicles improve recovery in a porcine model of ischemic stroke. *Stroke* 49 1248–1256. 10.1161/strokeaha.117.020353 29650593 PMC5916046

[B263] Webb R. L. Kaiser E. E. Scoville S. L. Thompson T. A. Fatima S. Pandya C. (2018b). Human neural stem cell extracellular vesicles improve tissue and functional recovery in the murine thromboembolic stroke model. *Transl. Stroke Res.* 9 530–539. 10.1007/s12975-017-0599-2 29285679 PMC6132936

[B264] Webster M. J. Herman M. M. Kleinman J. E. Shannon Weickert C. (2006). BDNF and trkB mRNA expression in the hippocampus and temporal cortex during the human lifespan. *Gene Expr. Patterns* 6 941–951. 10.1016/j.modgep.2006.03.009 16713371

[B265] Wiklander O. P. B. Brennan M. Lötvall J. Breakefield X. O. El Andaloussi S. (2019). Advances in therapeutic applications of extracellular vesicles. *Sci. Transl. Med.* 11:eaav8521. 10.1126/scitranslmed.aav8521 31092696 PMC7104415

[B266] Wiklander O. P. B. Nordin J. Z. O’loughlin A. Gustafsson Y. Corso G. Mäger I. (2015). Extracellular vesicle in vivo biodistribution is determined by cell source, route of administration and targeting. *J. Extracell. Vesicles* 4:26316. 10.3402/jev.v4.26316 25899407 PMC4405624

[B267] Williams C. E. Gunn A. J. Synek B. Gluckman P. D. (1990). Delayed seizures occurring with hypoxic-ischemic encephalopathy in the fetal sheep. *Pediatr. Res.* 27 561–565. 10.1203/00006450-199006000-00004 2356099

[B268] Williams C. E. Gunn A. Gluckman P. D. (1991). Time course of intracellular edema and epileptiform activity following prenatal cerebral ischemia in sheep. *Stroke* 22 516–521. 10.1161/01.str.22.4.516 2024281

[B269] Willms E. Cabañas C. Mäger I. Wood M. J. A. Vader P. (2018). Extracellular vesicle heterogeneity: subpopulations, isolation techniques, and diverse functions in cancer progression. *Front. Immunol.* 9:738. 10.3389/fimmu.2018.00738 29760691 PMC5936763

[B270] Willms E. Johansson H. J. Mäger I. Lee Y. Blomberg K. E. M. Sadik M. (2016). Cells release subpopulations of exosomes with distinct molecular and biological properties. *Sci. Rep.* 6:22519. 10.1038/srep22519 PMC4773763 26931825

[B271] Wurzelmann M. Romeika J. Sun D. (2017). Therapeutic potential of brain-derived neurotrophic factor (BDNF) and a small molecular mimics of BDNF for traumatic brain injury. *Neural Regen. Res.* 12 7–12. 10.4103/1673-5374.198964 28250730 PMC5319242

[B272] Wyss-Coray T. Feng L. Masliah E. Ruppe M. D. Lee H. S. Toggas S. M. (1995). Increased central nervous system production of extracellular matrix components and development of hydrocephalus in transgenic mice overexpressing transforming growth factor-beta 1. *Am. J. Pathol.* 147 53–67. 7604885 PMC1869892

[B273] Xin D. Li T. Chu X. Ke H. Yu Z. Cao L. (2020). Mesenchymal stromal cell-derived extracellular vesicles modulate microglia/macrophage polarization and protect the brain against hypoxia-ischemic injury in neonatal mice by targeting delivery of miR-21a-5p. *Acta Biomater.* 113 597–613. 10.1016/j.actbio.2020.06.037 32619670

[B274] Xin H. Li Y. Buller B. Katakowski M. Zhang Y. Wang X. (2012). Exosome-mediated transfer of miR-133b from multipotent mesenchymal stromal cells to neural cells contributes to neurite outgrowth. *Stem Cells* 30 1556–1564. 10.1002/stem.1129 22605481 PMC3495063

[B275] Xin H. Li Y. Cui Y. Yang J. J. Zhang Z. G. Chopp M. (2013a). Systemic administration of exosomes released from mesenchymal stromal cells promote functional recovery and neurovascular plasticity after stroke in rats. *J. Cereb. Blood Flow Metab.* 33 1711–1715. 10.1038/jcbfm.2013.152 23963371 PMC3824189

[B276] Xin H. Li Y. Liu Z. Wang X. Shang X. Cui Y. (2013b). MiR-133b promotes neural plasticity and functional recovery after treatment of stroke with multipotent mesenchymal stromal cells in rats via transfer of exosome-enriched extracellular particles. *Stem Cells* 31 2737–2746. 10.1002/stem.1409 23630198 PMC3788061

[B277] Yan S. Zhang H. Xie W. Meng F. Zhang K. Jiang Y. (2017). Altered microRNA profiles in plasma exosomes from mesial temporal lobe epilepsy with hippocampal sclerosis. *Oncotarget* 8 4136–4146. 10.18632/oncotarget.13744 27926529 PMC5354818

[B278] Yang J. Zhang X. Chen X. Wang L. Yang G. (2017). Exosome mediated delivery of miR-124 promotes neurogenesis after ischemia. *Mol. Ther. Nucleic Acids* 7 278–287. 10.1016/j.omtn.2017.04.010 28624203 PMC5415550

[B279] Yao H. Ma R. Yang L. Hu G. Chen X. Duan M. (2014). MiR-9 promotes microglial activation by targeting MCPIP1. *Nat. Commun.* 5:4386. 10.1038/ncomms5386 PMC4104446 25019481

[B280] Yeo K. T. Lee Q. Y. Quek W. S. Wang Y. A. Bolisetty S. Lui K. (2015). Trends in morbidity and mortality of extremely preterm multiple gestation newborns. *Pediatrics* 136 263–271. 10.1542/peds.2014-4075 26169427

[B281] Yoon Y. J. Kim O. Y. Gho Y. S. (2014). Extracellular vesicles as emerging intercellular communicasomes. *BMB Rep.* 47 531–539. 10.5483/bmbrep.2014.47.10.164 25104400 PMC4261509

[B282] Zagrean A. M. Hermann D. M. Opris I. Zagrean L. Popa-Wagner A. (2018). Multicellular crosstalk between exosomes and the neurovascular unit after cerebral ischemia. Therapeutic implications. *Front. Neurosci.* 12:811. 10.3389/fnins.2018.00811 30459547 PMC6232510

[B283] Zhang G. Zhu Z. Wang H. Yu Y. Chen W. Waqas A. (2020). Exosomes derived from human neural stem cells stimulated by interferon gamma improve therapeutic ability in ischemic stroke model. *J. Adv. Res.* 24 435–445. 32551140 10.1016/j.jare.2020.05.017 PMC7289755

[B284] Zhang L. Graf I. Kuang Y. Zheng X. Haupt M. Majid A. (2021). Neural progenitor cell-derived extracellular vesicles enhance blood-brain barrier integrity by NF-κB (Nuclear Factor-κB)-dependent regulation of ABCB1 (ATP-binding cassette transporter B1) in stroke mice. *Arterioscler. Thromb. Vasc. Biol.* 41 1127–1145. 10.1161/atvbaha.120.315031 33327747 PMC7901534

[B285] Zhang Y. Chopp M. Meng Y. Katakowski M. Xin H. Mahmood A. (2015). Effect of exosomes derived from multipluripotent mesenchymal stromal cells on functional recovery and neurovascular plasticity in rats after traumatic brain injury. *J. Neurosurg.* 122 856–867. 10.3171/2014.11.jns14770 25594326 PMC4382456

[B286] Zwanenburg A. Jellema R. K. Jennekens W. Ophelders D. Vullings R. Van Hunnik A. (2013). Heart rate-mediated blood pressure control in preterm fetal sheep under normal and hypoxic-ischemic conditions. *Pediatr. Res.* 73 420–426. 10.1038/pr.2013.15 23340656

